# Lanthanide Ionic Radius Modulation for Tailored Triple-Cross-Linked Luminescent Gelatin/Alginate Hydrogels: Structural, Mechanical, and Sensing Insights

**DOI:** 10.1021/jacsau.5c00647

**Published:** 2025-08-13

**Authors:** Shu-Ying Wu, Yu-Ning An, Yi-Cheun Yeh

**Affiliations:** Institute of Polymer Science and Engineering, 33561 National Taiwan University, Taipei 10617, Taiwan

**Keywords:** hydrogel, lanthanide, luminescence, sensor

## Abstract

Lanthanide-containing hydrogels have emerged as a promising category of luminescent materials for sensing applications. However, a systematic investigation of lanthanide ions with varying ionic radii to reveal the structure–property–function relationships within the hydrogel network remains unexplored. This study integrates different lanthanide (Ln^3^^+^) ions (i.e., samarium (Sm^3^^+^), europium (Eu^3^^+^), or terbium (Tb^3^^+^)) into polymeric networks composed of phenylboronic acid-grafted polyethylenimine (PBA-PEI)-modified gelatin (PPG) and alginate-dialdehyde (ADA), resulting in triple-cross-linked PPG/ADA-Ln^3^^+^ hydrogels that are stabilized via dynamic bonds. The influence of lanthanide ionic radius on the microstructures, properties (i.e., luminescence, rheological behavior, mechanical strength, swelling capacity, and stability), and sensing performance is systematically investigated. PPG/ADA-Ln^3^^+^ lyophilized hydrogels demonstrate a remarkable ability to differentiate acidic and basic vapors in volatile organic compounds through linear discriminant analysis (LDA), and this capability is further explored for bacterial differentiation. Overall, smaller Tb^3^^+^ ions induce the formation of denser networks with enhanced mechanical properties and contribute superior sensing capabilities to the hydrogel, attributed to optimized coordination with the carboxylate groups of the polymers. This comprehensive study elucidates the critical role of lanthanide ionic radius in shaping the structural and functional attributes of PPG/ADA-Ln^3^^+^ hydrogels, showing their potential as versatile biomaterials with tailored properties for a wide range of applications.

## Graphical abstract

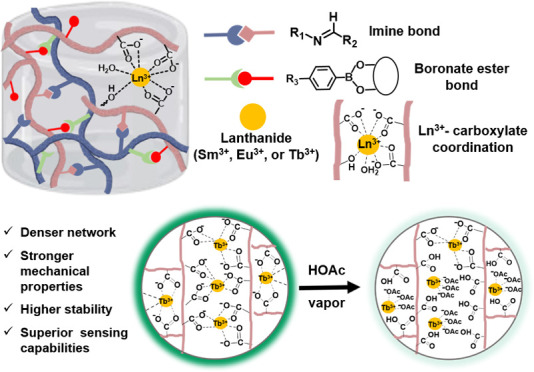

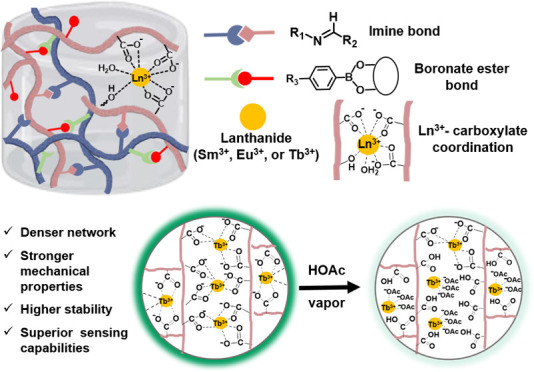

## 1 Introduction

Lanthanide-containing hydrogels have received significant attention due to their exceptional luminescent properties of high quantum efficiency, excellent color purity, great photostability, large Stokes shift, and long lifetime.^1–6^ Therefore, lanthanide-containing hydrogels have found extensive applications across various fields, such as tissue engineering,^7^ biosensing,^8^ 3D printing,^9^ and controlled drug release.^10^ Luminophores of lanthanide ions,^11^ lanthanide complexes,^12,13^ and lanthanide-incorporated nanomaterials (e.g., metal–organic frameworks (MOFs),^14^ upconverting nanorods,^15^ inorganic nanotubes,^16^ and laponites^17,18^ ) are frequently used as light-emitting sources in the lanthanide-containing hydrogel network. In particular, the small-sized lanthanide ions allow them to be simply hybridized with polymers at the molecular level. Integrating different types of lanthanide ions into the functionalized polymeric networks through coordination assembly is a versatile strategy to fabricate hydrogels with various structures and properties. For example, Wang et al. developed a multiluminescent hydrogel via noncovalent interactions using poly­(vinyl alcohol) (PVA), poly­(acrylic amide-*co*-2-acrylamido-2-methylpropanesulfonic acid) P­(AM/AMPS), and lanthanide ions (i.e., europium (Eu^3+^) and terbium (Tb^3+^)).^19^ Sun et al. constructed a three-dimensional network by using a rigid lamellar structure of poly­(dodecylglyceryl itaconate) (pDGI) formed by shear flow-induced self-assembly, as well as incorporating a polymerized structure of acrylamide (AAm) and N,N’-Dimethylacrylamide (DMA).^20^ Furthermore, lanthanide ions (i.e., Eu^3+^ or Tb^3+^) were introduced to generate the coordination bonds with the fluorescent monomer 6-acrylamidopicolinate (6APA) in the pDGI/p­(AAm-DMA-6APA) hydrogel network, forming multicolored fluorescent hydrogels. Wei et al. reported a new class of bioinspired polymeric hydrogels by radical copolymerization of potassium 6-acrylamidopicolinate (K6APA) and *N*-isopropylacrylamide (NIPAM), followed by the coordination with Eu^3+^ or Tb^3+^ ions, where the emission color can be adjusted by changes in acidity or alkalinity.^21^

Lanthanide ions have been demonstrated to not only impart diverse luminescence characteristics to the hydrogels but also improve and modulate their mechanical properties. For example, Wang et al. utilized lanthanide (Ln^3+^) ions (i.e., Eu^3+^, Tb^3+^, or Eu^3+^/Tb^3+^) as both luminescent emitters and physical cross-linkers to provide a stronger interaction with alginate polymers than sodium (Na^+^) ions in the alginate/polyacrylamide network.^22^ The tough Ln-alginate/polyacrylamide hydrogel presented tensile strength of ∼1 MPa, compressive strength of ∼3.4 MPa, and energy dissipation of ∼10^4^ kJ m^–3^. In another example, He et al. added Ln^3+^ ions (i.e., Tb^3+^ and dysprosium (Dy^3+^)) to improve the network strength of sodium alginate/polyethylene glycol/polyacrylamide (SA/PEG/PAM), showing a 132.8% increase in compressive properties of the hydrogels after doping Ln^3+^ ions.^23^ The study also revealed that a higher Tb/Dy ratio in the network offered better mechanical properties to the hydrogel, resulting from the small radius of the Tb^3+^ ions compared to that of the Dy^3+^ ions, as the gravitational force between the electrons with a small ion and the matrix molecules is larger. Xu et al. incorporated lanthanide organic complexes (Ln-L) into the polyacrylamide/hypromellose (PAM/HPMC) network, where stronger mechanical properties of the Tb-L containing network compared to the Eu-L containing network were observed due to the smaller ionic radius of the Tb^3+^ ion compared to the Eu^3+^ ion.^24^ This size difference enabled superior stress–strain performance at the same spatial site resistance of Tb^3+^ ions to result in a smaller and more intense intermolecular cross-linking effect.

The luminescent lanthanide-containing hydrogels are highly attractive in sensing applications, such as pH,^25,26^ temperature,^26^ human motion,^19^ metal ions,^27^ volatile organic compounds (VOCs),^28,29^ and bacteria.^30^ For example, Yang et al. prepared a smart hydrogel by using Eu polyoxometalate (EuPOM) and poly­(2-acrylamido-2-methyl-1-propanesulfonicacid) (PAMPSA).^25^ After being treated with ammonia (NH_3_) vapor, the EuPOM/PAMPSA hydrogel emitted red light, which came from ligand-to-metal charge transfer (LMCT); however, when treated with hydrochloric acid (HCl) vapor, the red luminescence disappeared due to the energy transfer failing by protonated hydrogen bond among EuPOM and PAMPSA. Su et al. fabricated a series of luminescent lanthanide-containing hydrogels for VOC differentiation, showing that the exposure to acetic acid (HOAc), NH_3_, and formaldehyde (FA) vapor led to quenching of the luminescence of the hydrogel.^28^ The quenching of the luminescence was caused by protonation of the bonding between lanthanides and ligands or competing interactions that impacted the antenna effect. Zhou et al. prepared a pH hydrogel sensor made from chitosan/dextran aldehyde/lanthanide coordination polymer (CS/DEX/CP), where the color change of luminescence caused by bacterial growth can be observed with the naked eye.^30^ In our previous work, the hydrogel made from phenylboronic acid-grafted polyethylenimine (PBA–PEI)-modified gelatin (PPG), alginate dialdehyde (ADA), and Eu^3+^ ions, demonstrated the ability to monitor bacterial growth through the quenching of the luminescence caused by the gas product of bacterial metabolism.^31^

The use of various lanthanide ions to modulate the structures and properties of luminescent lanthanide-containing hydrogels has been well demonstrated. However, to the best of our knowledge, a systematic investigation of lanthanide ions with varying ionic radii to elucidate the structure–property–function relationships within the hydrogel network remains unexplored. Understanding the structure–property–function relationships of the lanthanide-containing hydrogels is fundamental to show that the changes at the molecule- or network-level influence key material properties, which in turn govern their functional performance in real-world applications. A comprehensive investigation of how structural variations affect properties and functionalities enables predictive material engineering, moving beyond trial-and-error approaches. Moreover, the structure–property–function insight allows for precisely tailoring luminescent lanthanide-containing hydrogels to meet specific application requirements across diverse fields such as bioimaging, sensing, and anticounterfeiting.

Here, we aim to integrate lanthanide ions with varying ionic radii into the polymeric network to reveal their potential in fine-tuning the structures and properties of hydrogels as well as in sensing applications. In our design, different lanthanide ions (i.e., samarium (Sm^3+^), Eu^3+^, or Tb^3+^) were introduced to the polymeric networks of PPG and ADA to fabricate PPG/ADA-Ln^3+^ hydrogels through a dual-channel syringe (Scheme 1). These three luminescent lanthanide ions offer distinct advantages over nonluminescent lanthanides and other hard Lewis acid ions, owing to their sharp, well-defined emission profiles and exceptionally long luminescence lifetimes. Combined with their inherent chemical and photostability, these features make them highly effective optical probes for various advanced analytical and imaging applications.^32,33^ Notably, each luminescent lanthanide ion exhibits characteristic emission colors (e.g., Sm^3+^ emits orange-red, Eu^3+^ emits red, and Tb^3+^ emits green), enabling multiplexed detection through wavelength-selective analysis. This spectral diversity facilitates multicolor imaging^34,35^ and the simultaneous identification of multiple analytes within complex systems.^36^

**Scheme 1:**
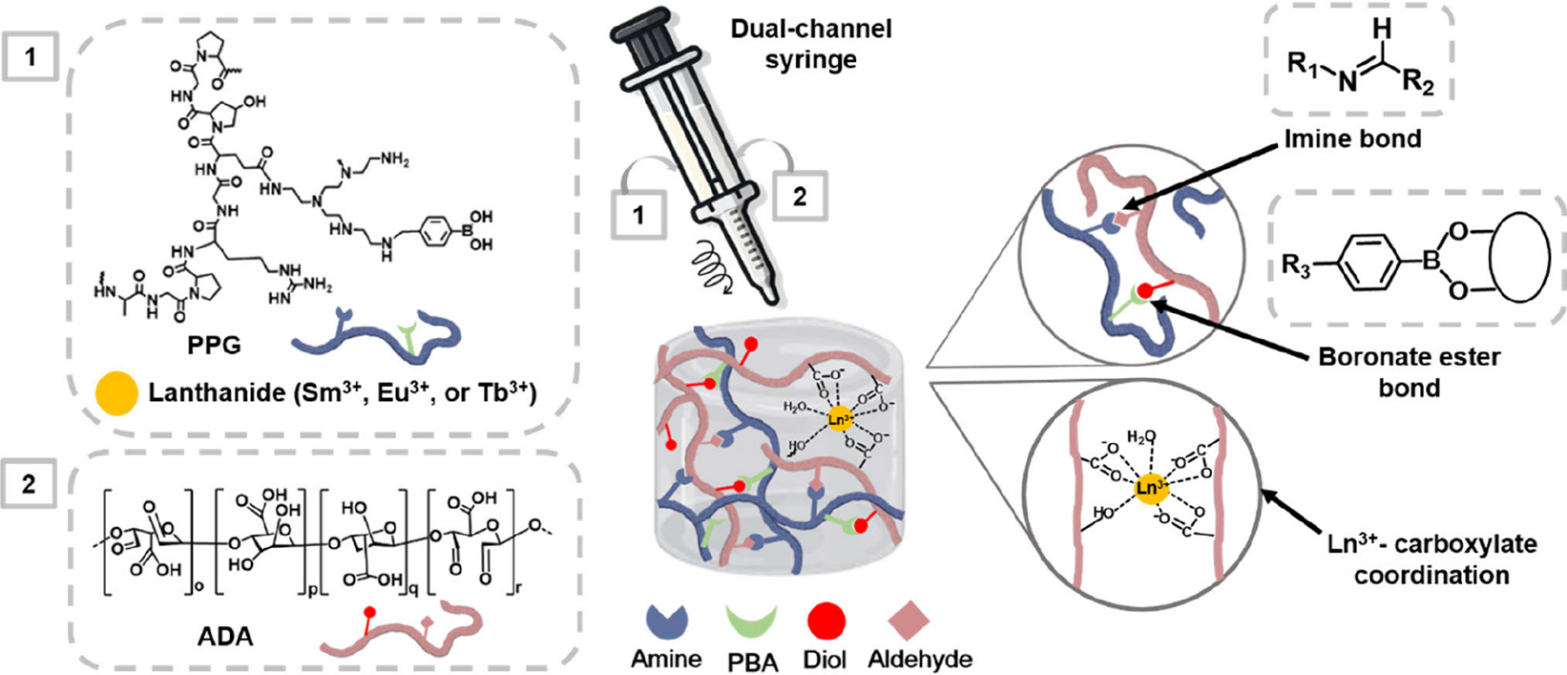
Schematic Illustrations of the Chemical Structures of PPG and ADA, as Well as the PPG/ADA-Ln^3+^ Hydrogel Formation through the Three Crosslinking Mechanisms

In our previous study,^31^ lanthanide ions were incorporated into the hydrogel network through sequential freezing-drying-swelling (FDS) and freezing-thawing (FT) cycles, aiming to enhance luminescence and network stability. However, this method posed challenges in precisely controlling the lanthanide content within the hydrogel. To address this, we adopted a direct incorporation approach during hydrogel synthesis in this study, enabling consistent and equivalent loading of the three lanthanide ions for accurate structural and property comparisons.

PPG/ADA-Ln^3+^ hydrogels possessed three types of dynamic cross-links in the network, including imine, boronate ester, and lanthanide-carboxylate bonds. The role of the lanthanide ion radius on microstructures and properties (i.e., luminescence, rheological behavior, mechanical strength, swelling capacity, self-healing efficiency, and stability), as well as the potential applications of PPG/ADA-Ln^3+^ hydrogels in distinguishing VOCs and bacteria, were systematically investigated.

## 2 Results and Discussion

### 2.1 Syntheses and Characterizations of Hydrogels

ADA and PPG were synthesized based on our previous work.^37^ Briefly, alginate was oxidized by sodium periodate to obtain ADA with an oxidation degree of 15% and an aldehyde amount of 1.5 mmol/g. The weight-average molecular weight (*M*_w_) of ADA was ∼130 kDa with a polydispersity index (PDI) of 1.05 compared to alginate (*M*_w_ = ∼165 kDa, PDI = 1.06). The ^1^H nuclear magnetic resonance (NMR) spectroscopy was further used to determine the ratio between 1 and 4 linked β-D-mannuronic acid (M) and 1–4 linked α-L-guluronic acid (G) of ADA based on the reported calculation method.^38–40^ By comparing the relative integral areas of peaks A, B, and C in the NMR spectrum of ADA, the M/G ratio of ADA was 1.20 (Figure S1). Phenylboronic acid-grafted polyethylenimine (PBA-PEI) was conjugated to gelatin to generate PBA-PEI-grafted gelatin (PPG) through amide coupling. The *M*_w_ of PPG was ∼130 kDa with a PDI of 1.13 compared to gelatin (*M*_w_ = ∼72 kDa, PDI = 1.15).

When mixing PPG, ADA, and Ln^3+^ ions, the amines of PPG reacted with the aldehydes of ADA, the phenylboronic acids of PPG interacted with the diols of ADA, and Ln^3+^ ions coordinated with carboxylate groups of polymers, leading to the spontaneous formation of imine, boronate ester, and lanthanide-carboxylate bonds within the polymeric network of the PPG/ADA-Ln^3+^ hydrogels, respectively. Different lanthanide ions with various radii (i.e., Sm^3+^ (0.964 Å), Eu^3+^ (0.950 Å), or Tb^3+^ (0.923 Å)^41^ were incorporated into the PPG/ADA-Ln^3+^ hydrogels.

X-ray photoelectron spectroscopy (XPS) was used to analyze the chemical bonding in the hydrogel structure, where the hydrogels were freeze-dried before measurement. In the XPS spectrum of PPG/ADA lyophilized hydrogel, only carbon (C), nitrogen (N), and oxygen (O) were detected at 288, 421, and 530 eV, respectively (Figure S2a). When lanthanides were presented in the PPG/ADA-Ln^3+^ network, the new peaks of lanthanides were detected at the characteristic binding energy range of 1084–1277 eV (Figure S2b–d). The peaks of Sm 3d_3/2_, Sm 3d_5/2_, Eu 3d_3/2_, Eu 3d_5/2_, Tb 3d_3/2_, and Tb 3d_5/2_ were found at 1110.5, 1183.6, 1165.2, 1135.0, 1276.9, and 1242.3 eV, respectively (Figure 1a–c). The four curve-fitted peaks in the deconvoluted O 1s spectra for PPG/ADA and PPG/ADA-Ln^3+^ lyophilized hydrogels indicated the different chemical states of oxygen atoms (Figures 1d–f and S3). For example, in the O 1s deconvoluted spectrum of PPG/ADA-Sm^3+^ lyophilized hydrogel, the peaks at 530.9, 532.0, 532.9, and 536.0 eV corresponded to C=O, Sm–O, C–O, and B–O, respectively (Figure 1d). Quantitative integration of the XPS peak areas corresponding to Ln-O bonds revealed that the Tb–O peak (174,900) was significantly greater than those of Sm–O (74,800) and Eu–O (129,800) (Table S1). Since the XPS peak area is directly related to the relative atomic concentration, the pronounced Tb–O signal suggests a higher degree of coordination between Tb^3^^+^ ions and oxygen, compared to Sm^3^^+^ and Eu^3^^+^ ions.^42,43^ Besides, researchers have employed computational calculations to investigate the strength of Ln–O bonds.^44^ For example, Solov’ev et al. used linear free energy relationships (LFER) and several machine learning methods (i.e., multiple linear regression (MLR), support vector machine (SVM), artificial neural networks (ANN), and K-nearest neighbor regression (kNN)) to describe the stability of complexes of lanthanide ions (from cerium (Ce^3+^) to Lutetium (Lu^3+^)) with organic ligands (e.g., carboxylate, amino acids, and cyclic polydentate ligands) in water.^45^ The results showed that heavier (smaller) lanthanide ions exhibited stronger interactions with the carboxylate ligand, owing to the greater stability of their complexes.

**Figure 1:**
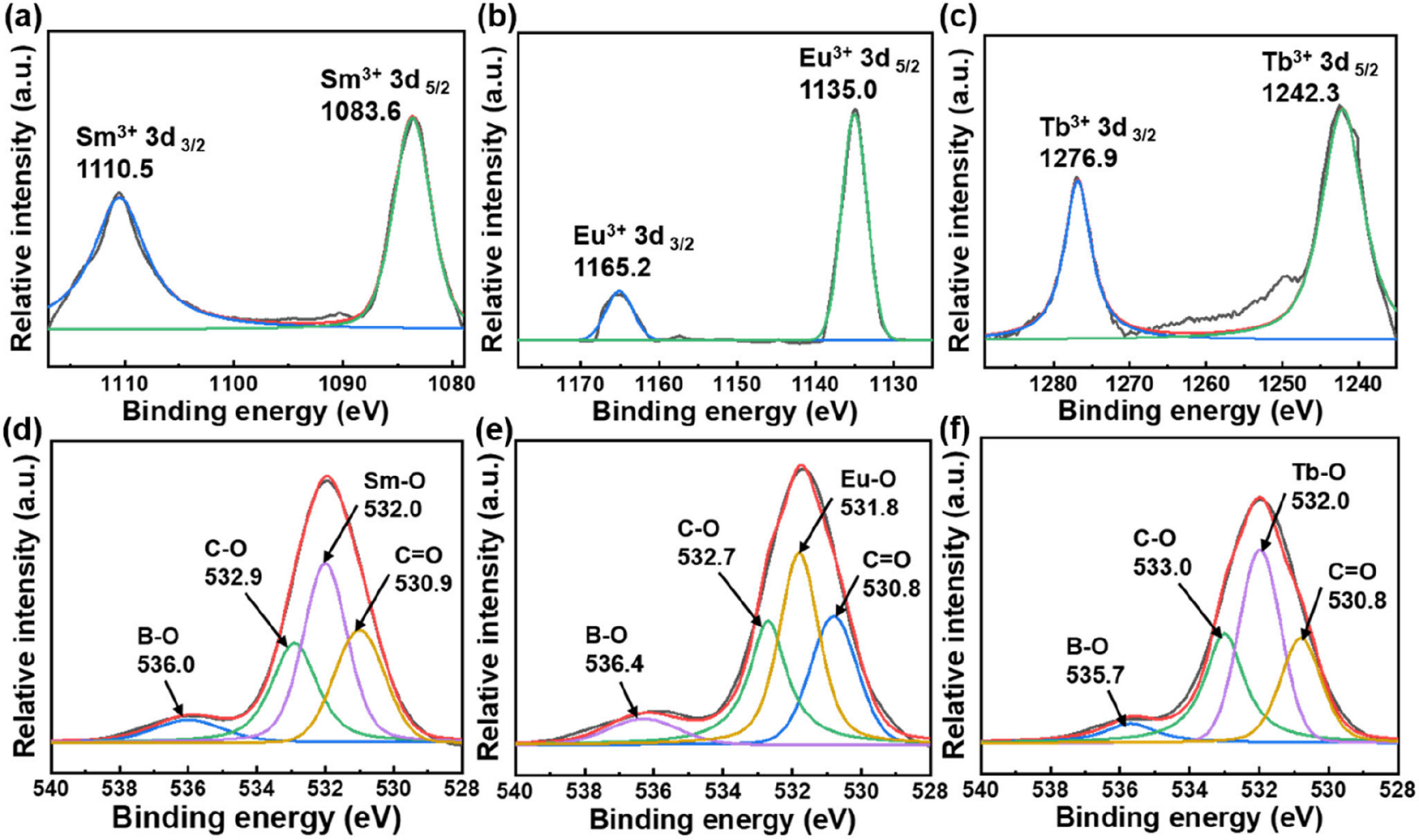
XPS spectra of different lanthanide ions for (a) PPG/ADA-Sm^3+^, (b)­PPG/ADA-Eu^3+^, and (c) PPG/ADA-Tb^3+^ lyophilized hydrogels. O 1s deconvoluted XPS spectra for (d) PPG/ADA-Sm^3+^, (e) PPG/ADA-Eu^3+^, and (f) PPG/ADA-Tb^3+^ lyophilized hydrogels.

On the other hand, the C 1s deconvoluted spectra of PPG/ADA and PPG/ADA-Ln^3+^ lyophilized hydrogels showed five peaks (Figure S4), with 284.4, 285.2, 285.4, 286.3, and 287.9 eV assigned to the C–C/C–H, C–OH/C=N, C–N, O–C=O, and C–B bonds in the PPG/ADA-Sm^3+^ lyophilized hydrogel, respectively (Figure S4b). In the N 1s deconvoluted spectra of the PPG/ADA and PPG/ADA-Ln^3+^ lyophilized hydrogels (Figure S5), three peaks at 398.5, 399.7, and 400.3 eV were attributed to N=C, NH_2_, and N–H/N–C bonds in the PPG/ADA-Sm^3+^ lyophilized hydrogel, respectively (Figure S5b).

In the fluorescent spectrum of PPG/ADA hydrogel, a broad band in the range of 450–550 nm was observed under excitation at 360 nm, and the intensity of the fluorescence was enhanced after the hydrogel was lyophilized (Figure S6a). The PPG/ADA-Ln^3+^ hydrogels also mainly showed the fluorescence contributed from the polymers (Figure 2a–c), as the water molecules can quench the luminescence of lanthanide ions in the network through their high-energy vibrational modes.^46^ After the freeze-drying process, several characteristic luminescence peaks were shown in the spectra of PPG/ADA-Ln^3+^ lyophilized hydrogels (Figure 2a–c). The luminescence spectrum of PPG/ADA-Sm^3+^ lyophilized hydrogel showed four distinct luminescent peaks at 561, 598, 644, and 706 nm, corresponding to the transitions of ^4^G_5/2_ → ^6^H_5/2_, ^4^G_5/2_ → ^6^H_7/2_, ^4^G_5/2_ → ^6^H_9/2_, and ^4^G_5/2_ → ^6^H_11/2_, respectively (Figure 2a). PPG/ADA-Eu^3+^ lyophilized hydrogel presented luminescence peaks at 579, 591, 611, 650, and 700 nm, corresponding to the transitions of ^5^D_0_ → ^7^F_0_, ^5^D_0_ → ^7^F_1_, ^5^D_0_ → ^7^F_2_, ^5^D_0_ → ^7^F_3,_ and ^5^D_0_ → ^7^F_4_, respectively (Figure 2b). The peaks at 490, 544, 585, and 622 nm in the spectrum of PPG/ADA-Tb^3+^ lyophilized hydrogel corresponded to the transitions of ^4^G_5/2_ → ^6^H_6/2_, ^4^G_5/2_ → ^6^H_5/2_, ^4^G_5/2_ → ^6^H_4/2_, and ^4^G_5/2_ → ^6^H_3/2_, respectively (Figure 2c).

**Figure 2:**
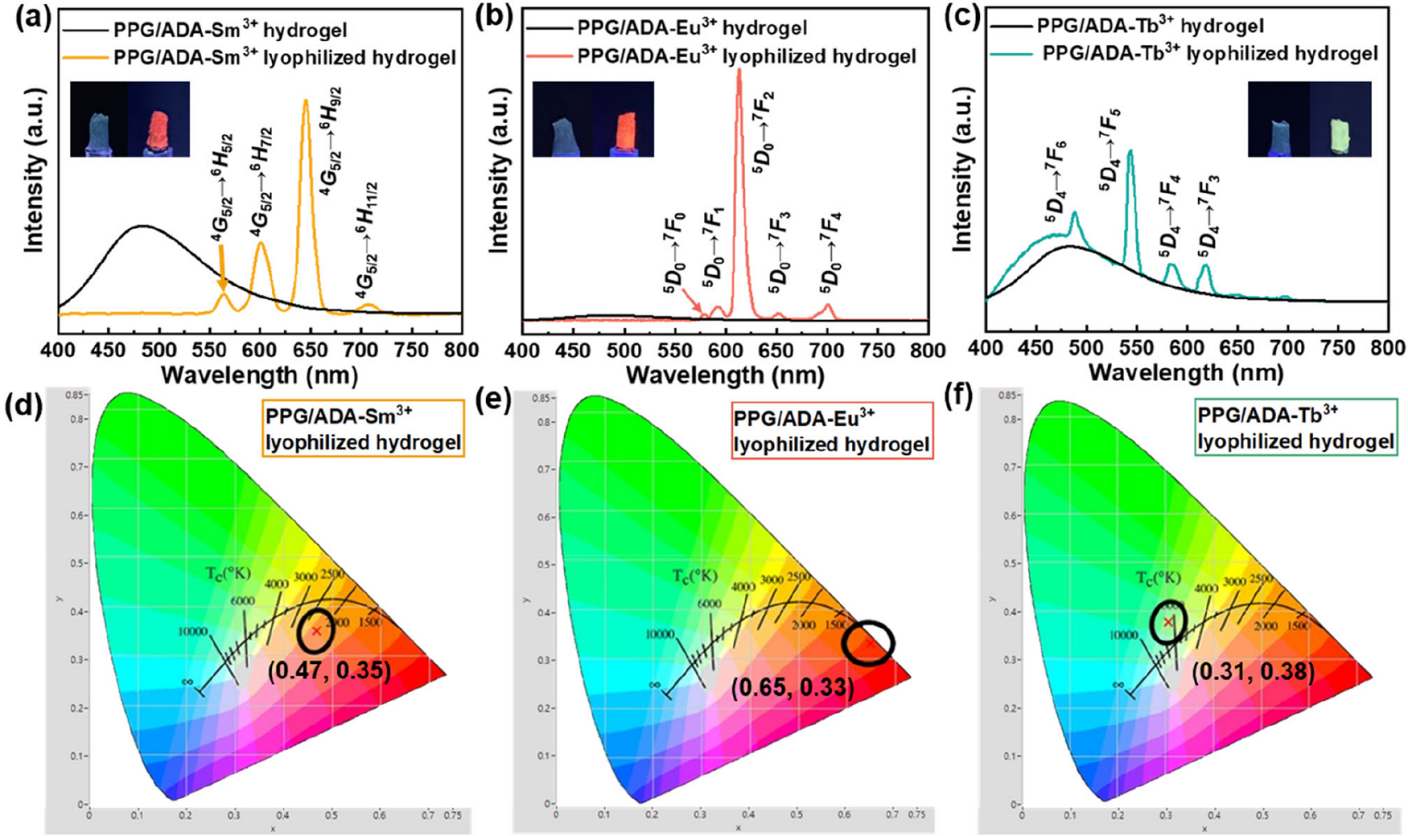
Luminescence spectra of (a) PPG/ADA-Sm^3+^, (b) PPG/ADA-Eu^3+^, and (c) PPG/ADA-Tb^3+^ lyophilized hydrogels. (Excitation wavelength (λ_ex_) = 360 nm) CIE images of (d) PPG/ADA-Sm^3+^, (e) PPG/ADA-Eu^3+^, and (f) PPG/ADA-Tb^3+^ lyophilized hydrogels (λ_ex_ = 360 nm). Inset: images of hydrogel (left) and lyophilized hydrogel (right) under UV light (365 nm).

The luminescence spectra of PPG/ADA and PPG/ADA-Ln^3+^ lyophilized hydrogels were converted into Commission internationale de l’éclairage (CIE) by using the photoluminescence instrument under excitation at 360 nm (Figures 2d–f and S6b). The color in the CIE corresponded to the appearance of lyophilized hydrogels under UV light irradiation, showing blue, orange, red, and green for PPG/ADA, PPG/ADA-Sm^3+^, PPG/ADA-Eu^3+^, and PPG/ADA-Tb^3+^ lyophilized hydrogels, respectively. Additionally, the photoluminescence quantum yield (PLQY) of PPG/ADA and PPG/ADA-Ln^3+^ lyophilized hydrogels was calculated through the following eq 1):

$$\mathrm{PLQY}=\frac{\mathrm{The}\;\mathrm{number}\;\mathrm{of}\;\mathrm{photons}\;\mathrm{emitted}}{\mathrm{The}\;\mathrm{number}\;\mathrm{of}\;\mathrm{photons}\;\mathrm{absorbed}}=\frac{E_{c}-E_{a}}{L_{a}-L_{c}}$$ 1

Where *E*_a_ and E_c_ are the integrated luminescence measured from empty and with the sample in the integrating sphere under 360 nm excitation. *L*_a_ and *L*_c_ are the integrated excitation profiles with and without the sample, respectively, when directly excited by the incident beam. The PLQY values of PPG/ADA, PPG/ADA-Sm^3+^, PPG/ADA-Eu^3+^, and PPG/ADA-Tb^3+^ lyophilized hydrogels were 0.20, 0.24, 3.85, and 0.23%, respectively.

### 2.2 Microstructures and Properties of Hydrogels

Scanning electron microscopy (SEM) was used to reveal the porous structures of lyophilized hydrogels (Figure 3a), showing the average pore sizes of PPG/ADA, PPG/ADA-Sm^3+^, PPG/ADA-Eu^3+^, and PPG/ADA-Tb^3+^ lyophilized hydrogels were ∼90.07, 81.17, 74.23, and 68.90 μm, respectively (Figure 3b). Microcomputed tomography (micro-CT) was further used to provide detailed information about the microstructures inside the hydrogels. The micro-CT images of lyophilized hydrogels showed relative pore size, blue for large, green for medium, and red for small pore size (Figure 3c). The pore sizes of PPG/ADA, PPG/ADA-Sm^3+^, PPG/ADA-Eu^3+^, and PPG/ADA-Tb^3+^ lyophilized hydrogels determined by micro-CT were 184.88, 168.12, 159.62, and 147.81 μm, respectively (Table S2). The total porosities of PPG/ADA, PPG/ADA-Sm^3+^, PPG/ADA-Eu^3+^, and PPG/ADA-Tb^3+^ lyophilized hydrogels were 88.53, 81.85, 82.71, and 89.76%, respectively. Micro-CT also showed the volume distributions of the hydrogels, revealing that the PPG/ADA lyophilized hydrogel presented the largest pore size and PPG/ADA-Tb^3+^ lyophilized hydrogel possessed the smallest pore size among the tested lyophilized hydrogels (Figure 3d). The pore size analyses of lyophilized hydrogels through micro-CT agreed with the results from the SEM images. According to the analyses of SEM and micro-CT images, PPG/ADA-Ln^3+^ lyophilized hydrogels generally possessed smaller pore sizes than the PPG/ADA lyophilized hydrogel, which should be due to the third Ln^3+^-carboxylate coordination cross-links to increase the cross-linking density of the network. Besides, the average pore sizes of PPG/ADA-Ln^3+^ lyophilized hydrogels were decreased along with smaller radii of lanthanide ions.

**Figure 3:**
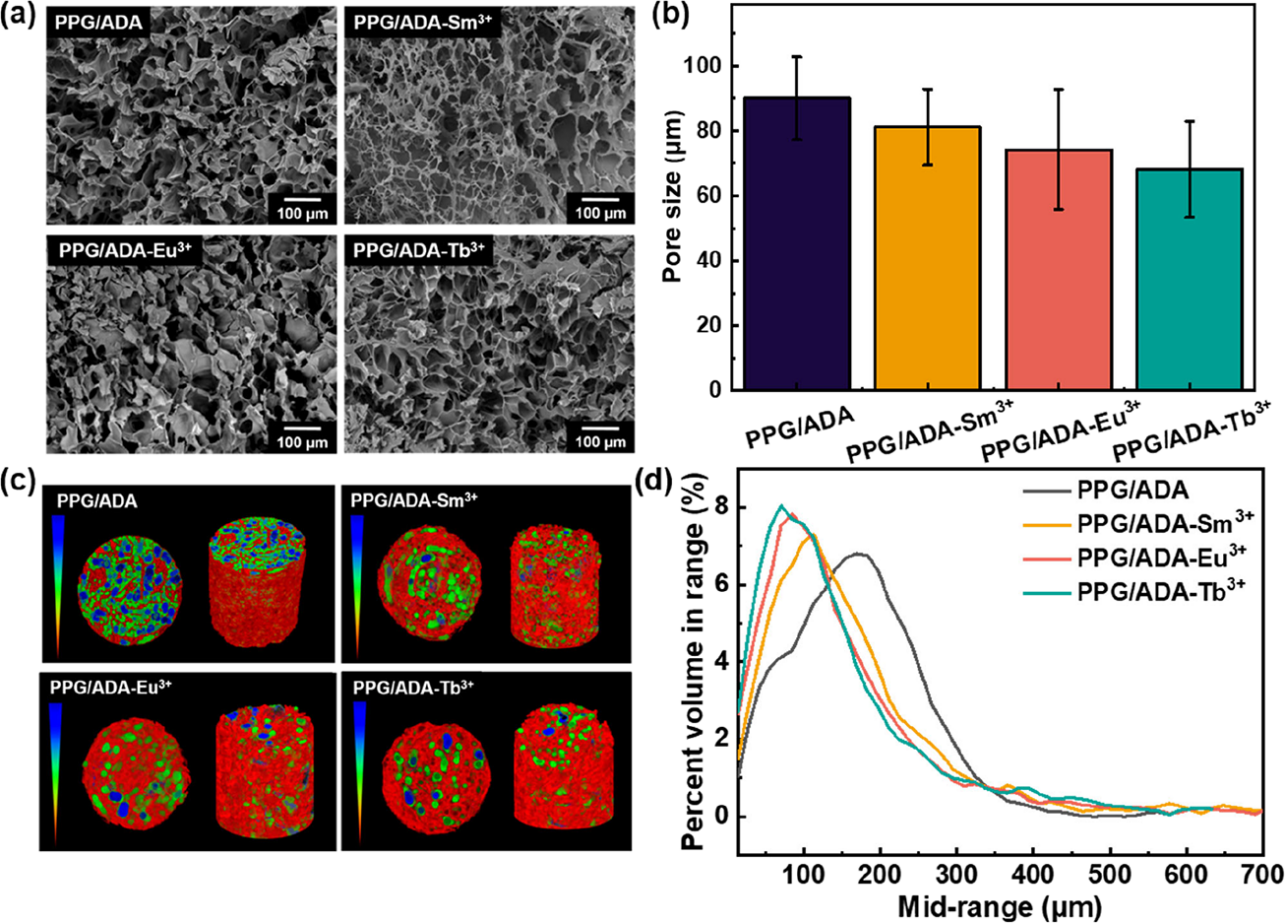
(a) SEM images and (b) pore size distributions of lyophilized hydrogels. (c) Micro-CT images and (d) percent volume range of lyophilized hydrogels.

The difference between the microstructures of the PPG/ADA-Ln^3+^ lyophilized hydrogels could be explained by the Ln^3+^-carboxylate binding affinity, which is usually influenced by the ionic radius.^47,48^ A smaller ionic radius increases charge density, enhancing the electrostatic interactions and coordination between metal ions and carboxylate groups. Given that the ionic radius of Tb^3^^+^ (0.923 Å) is smaller than that of Eu^3^^+^ (0.950 Å) and Sm^3^^+^ (0.964 Å),^41^ Tb^3^^+^ ions formed stronger interactions with the carboxylate groups in the network. In addition, the small ionic radius of Tb^3+^ ions might result in more Tb^3+^ ions participating in the Ln^3+^-carboxylate coordination compared to the larger-sized Eu^3^^+^ and Sm^3^^+^ ions.^49^ Overall, the optimized Tb^3^^+^-carboxylate bonding contributes to the increased network density observed in PPG/ADA-Tb^3^^+^ hydrogel compared to PPG/ADA-Sm^3^^+^ and PPG/ADA-Eu^3^^+^ hydrogels.

The effect of the radii of lanthanide ions on the rheological properties of PPG/ADA-Ln^3+^ hydrogels was investigated. In the time sweep test, PPG/ADA, PPG/ADA-Sm^3+^, PPG/ADA-Eu^3+^, and PPG/ADA-Tb^3^ hydrogels all showed stability within 6 min (Figure 4a). In the oscillation strain sweep, the storage modulus (*G*′) of PPG/ADA, PPG/ADA-Sm^3+^, PPG/ADA-Eu^3+^, and PPG/ADA-Tb^3^ hydrogels were ∼0.41, 7.94, 18.38, and 29.14 kPa, respectively (Figure 4b and Table S3). The cross-linking density of hydrogels can be further calculated through the eq 2):

$$G^{\prime }=vRT$$ 2

where *G*′ represents storage modulus in Pa, ν represents cross-linking density in mol/m^3^, *R* represents gas constant (8.314 J K^–1^ mol^–1^), and *T* represents the temperature in 298.15 K. Through the calculation, the cross-linking densities of the PPG/ADA, PPG/ADA-Sm^3+^, PPG/ADA-Eu^3+^, and PPG/ADA-Tb^3+^ hydrogels were ∼0.17, 3.37, 8.06, and 13.25 mol/m^3^, respectively.

**Figure 4:**
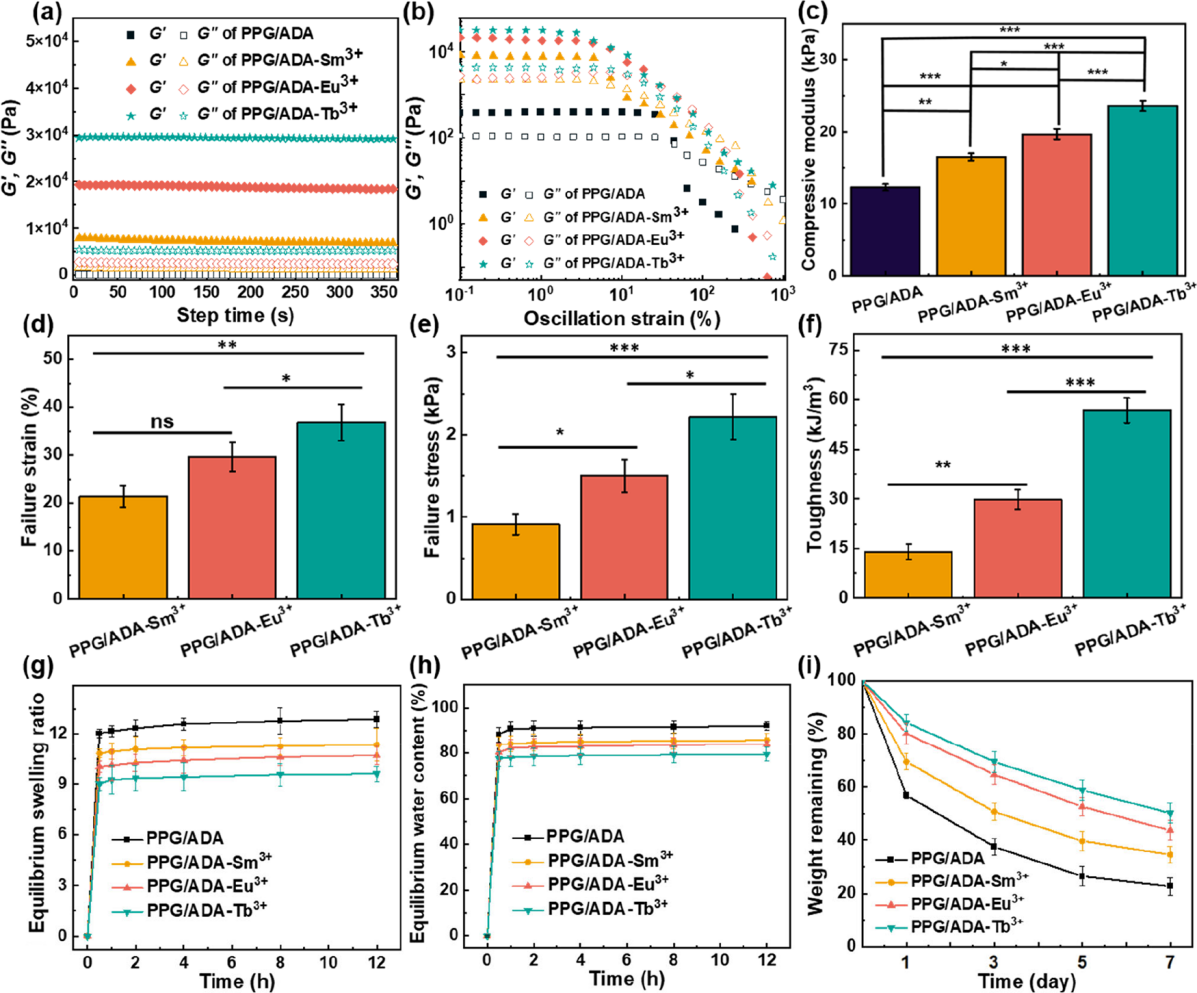
(a) Oscillation time sweep, (b) oscillation strain sweep, (c) compressive modulus, (d) failure strain, (e) failure stress, (f) toughness, (g) equilibrium swelling ratio, (h) equilibrium water content, and (i) weight remaining of hydrogels. The significance was set as *p* < 0.05 with *, **, and *** indicating *p* < 0.05, <0.01, and <0.001, respectively.

Stress relaxation experiments on the PPG/ADA-Ln^3^^+^ hydrogels were conducted by applying a constant strain while monitoring the resulting stress over time.^50^ All hydrogels exhibited an exponential decay in stress, a characteristic of viscoelastic behavior (Figure S7). Notably, the PPG/ADA-Tb^3+^ hydrogel exhibited the highest initial stress response, further supporting that smaller ionic cross-linkers enhanced the mechanical performance of the hydrogel network.

On the other hand, the compressive modulus of the PPG/ADA, PPG/ADA-Sm^3+^, PPG/ADA-Eu^3+^, and PPG/ADA-Tb^3+^ hydrogels were ∼12, 17, 20, and 23 kPa, respectively (Figure 4c), showing a similar trend with the rheological test. Tensile testing was further conducted to evaluate the failure strain, failure stress, and toughness of the hydrogels. Given the inherently weak mechanical properties of the PPG/ADA hydrogel, it could not be mounted securely using the testing clamps, rendering tensile measurement unfeasible. Therefore, tensile analysis was carried out on the three PPG/ADA-Ln^3+^ hydrogels (Figures 4d–f and S8), where the representative stress–strain curves of the hydrogels are shown in Figure S8b. The failure strains for the PPG/ADA-Sm^3+^, PPG/ADA-Eu^3+^, and PPG/ADA-Tb^3+^ hydrogels were ∼21.35, 29.62, and 36.80%, respectively (Figure 4d). The failure stresses for the PPG/ADA-Sm^3+^, PPG/ADA-Eu^3+^, and PPG/ADA-Tb^3+^ hydrogels were ∼0.85, 1.49, and 2.20 kPa, respectively (Figure 4e). The toughness values for the PPG/ADA-Sm^3+^, PPG/ADA-Eu^3+^, and PPG/ADA-Tb^3+^ hydrogels were ∼14.01, 29.75, and 56.82 kJ/m^3^, respectively (Figure 4f).

The rheological and mechanical properties of hydrogels were highly related to their microstructures. The large porous structure of the PPG/ADA hydrogel resulted in weaker mechanical strength compared to the PPG/ADA-Ln^3+^ hydrogels. On the other hand, the cross-linking densities of PPG/ADA-Ln^3+^ hydrogels can be tailored by selecting different lanthanide ions, with smaller Tb^3^^+^ ions exhibiting stronger Ln^3+^-carboxylate coordination to improve the mechanical strength of the hydrogels.

The swelling and degradation tests of the hydrogels were performed by immersing the lyophilized hydrogels in DI water at 25 °C. The swelling ratios of the PPG/ADA, PPG/ADA-Sm^3+^, PPG/ADA-Eu^3+^, and PPG/ADA-Tb^3+^ hydrogels were ∼13, 11, 10, and 9, respectively (Figure 4g). The water contents of the PPG/ADA, PPG/ADA-Sm^3+^, PPG/ADA-Eu^3+^, and PPG/ADA-Tb^3+^ hydrogels were ∼92, 86, 83, and 79%, respectively (Figure 4h). The weight remaining percentages of the PPG/ADA, PPG/ADA-Sm^3+^, PPG/ADA-Eu^3+^, and PPG/ADA-Tb^3+^ hydrogels were ∼23, 35, 44, and 50%, respectively, after immersion for 7 days (Figure 4i).

In general, hydrogels with larger pore sizes demonstrate improved water absorption and swelling capabilities. In this study, the PPG/ADA hydrogel with a large porous structure exhibited the highest swelling ratio and water content compared to the PPG/ADA-Ln^3+^ hydrogels. Additionally, within the PPG/ADA-Ln^3+^ hydrogels, the PPG/ADA-Sm^3+^ hydrogel with a looser network and a larger pore size showed a greater swelling ratio and water content than the PPG/ADA-Eu^3+^ and PPG/ADA-Tb^3+^ hydrogels. Regarding the stability during degradation tests, the PPG/ADA-Tb^3+^ hydrogel showed the best performance among the four hydrogels, which should be attributed to its robust network and high cross-linking density.

The self-healing properties of PPG/ADA and PPG/ADA-Ln^3+^ hydrogels were demonstrated through a cyclic strain sweep under 1% and 500% strain (Figure S9). When the strain came to 1%, the *G′* values were higher than the loss modulus (*G*″) values, showing the initial gel state of the samples. When the strain reached 500%, the *G*′ values were lower than the *G″* values, revealing the gel-to-sol transition. Due to the cyclic test, the strain was reduced to 1%, and the *G* *′* and *G″* values also returned to their initial state, demonstrating self-healing ability. The self-healing efficiency of hydrogels was further compared by determining the compressive modulus of the hydrogels that recovered from varying times (Figure S10). The self-healing efficiency of PPG/ADA hydrogel (∼71%) was higher than that of PPG/ADA-Sm^3+^ (∼62%), PPG/ADA-Eu^3+^ (∼59%), and PPG/ADA-Tb^3+^ (∼52%) hydrogels after 8 h. It has been reported that self-healing efficiency could be affected by cross-linking density.^51^ The hydrogel exhibited weak mechanical properties at a low cross-linking density, and the free volume allowed molecules to move easily to increase self-healing efficiency. Therefore, the PPG/ADA hydrogel with a loose cross-linked network possessed the superior self-healing efficiency among the tested hydrogels. However, after a longer self-healing time of 24 h, the self-healing efficiencies of all hydrogels were ∼90%, indicating the reach of equilibrium states.

The comparisons between PPG/ADA and PPG/ADA-Ln^3+^ hydrogels are illustrated in Scheme 2a,b. Given the lack of Ln^3+^-carboxylate coordination, PPG/ADA hydrogel showed a larger pore size, weaker compressive modulus, higher swelling ratio, lower weight remaining, and quicker self-healing efficiency compared to PPG/ADA-Ln^3+^ hydrogels. The role of different lanthanide ions in the microstructures and properties of PPG/ADA-Ln^3+^ hydrogels was further revealed in Scheme 2b. PPG/ADA-Tb^3+^ hydrogel exhibited smaller pore size, stronger compressive modulus, lower swelling ratio, higher weight remaining, and slower self-healing efficiency compared to PPG/ADA-Sm^3+^ and PPG/ADA-Eu^3+^ hydrogels. These superior properties of PPG/ADA-Tb^3+^ hydrogel should be attributed to the stronger binding affinity between Tb^3+^ ions and carboxylate groups, which is favored by the smaller ionic radius of Tb^3+^ (0.923 Å) ion compared to Sm^3+^ (0.964 Å) and Eu^3+^ (0.950 Å) ions.^47–49^ Meanwhile, the smaller radius could result in more Tb^3+^ ions participating in coordination. The effective participation of Tb^3+^ ions in coordination with carboxylate groups of the polymers improved the cross-linking density and resulted in a mechanically stronger network with a smaller pore size (Scheme 2c).

**Scheme 2:**
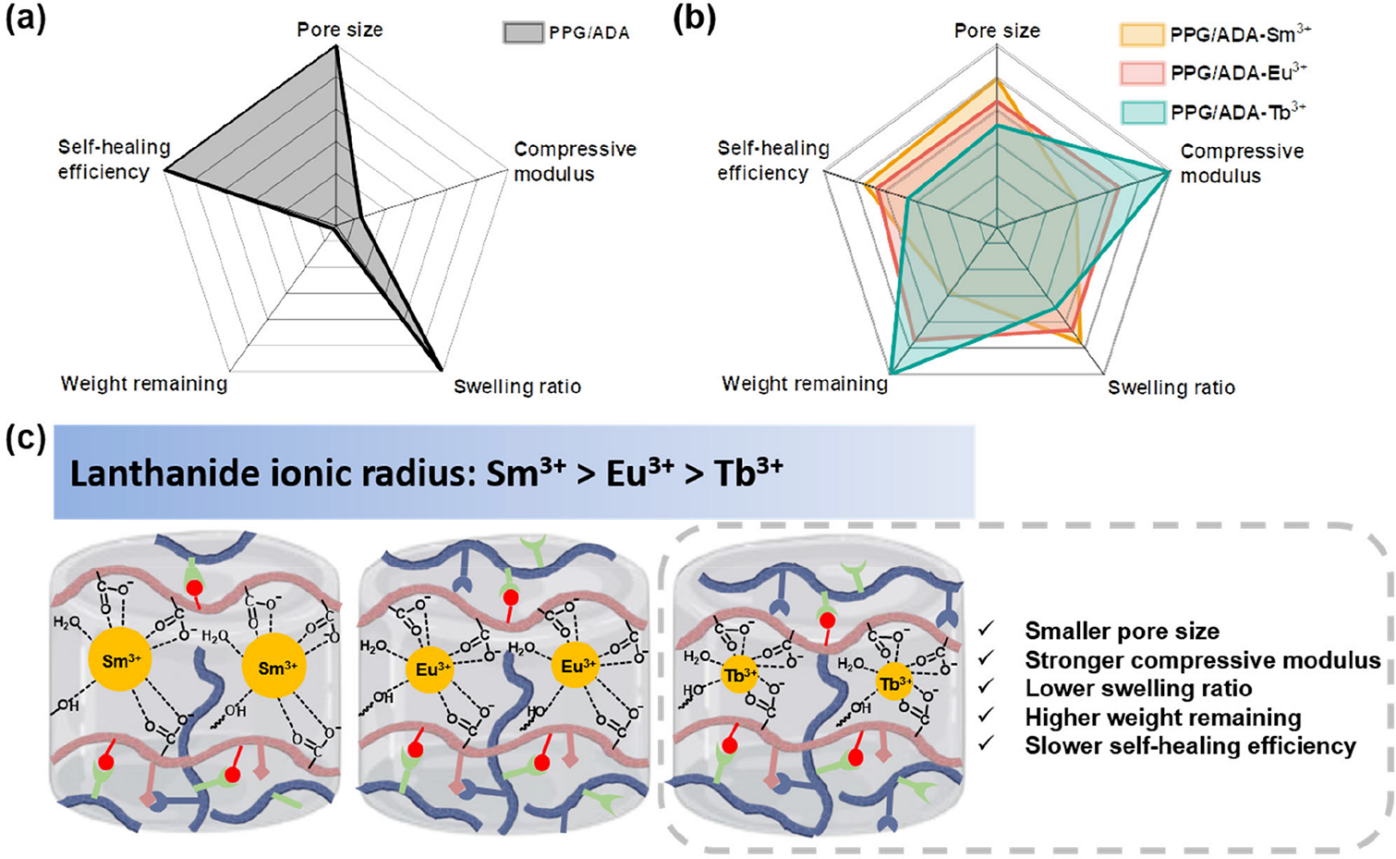
(a, b) Schematic Illustrations of the Comparisons between PPG/ADA and PPG/ADA-Ln^3+^ Hydrogels; (c) Schematic Illustrations of the Structural Changes of PPG/ADA-Ln^3+^ Hydrogels

In the triple cross-linked PPG/ADA-Ln^3+^ network (i.e., imine bond, borate ester bond, and Ln^3+^-carboxylate coordination), both imine bond and boronate ester bond exhibit dynamic exchange behavior, enabling reversible transimination^52^ or boronic ester exchange reactions^53^ that facilitate network rearrangement and reprocessability. For instance, an existing imine bond can undergo exchange with a free amine or aldehyde group, allowing for adaptive reconfiguration. However, while these dynamic covalent interactions contribute to self-healing and stress dissipation, their intrinsic reversibility might compromise mechanical robustness. In contrast, Ln^3+^-carboxylate coordination bonds are typically more thermodynamically stable and less labile under ambient conditions,^54^ enhancing the mechanical integrity and long-range structural cohesion of the network. Consequently, the combination of dynamic covalent bonds (i.e., imine and boronate ester) with more stable coordination bonds (i.e., Ln^3+^-carboxylate) yields a cross-linked hydrogel network capable of localized mobility, self-repair, and broader structural resilience. In particular, the coexistence of multiple dynamic bonds gives rise to complex equilibrium states and interconnected reaction pathways.^55–57^ By precisely tuning the relative kinetics of bond formation and dissociation, key material properties (e.g., self-healing efficiency, elasticity, and toughness) can be systematically optimized.^58^ Nevertheless, competitive binding effects among the functional groups should also be considered. Boronate ester and imine linkages are typically considered orthogonal in dynamic covalent chemistry, meaning they can form and break independently without cross-reactivity.^59–61^ Imine bond may compete with Ln^3+^ ions for primary amine coordination.^62–64^ Ln^3+^-carboxylate coordination, while potentially orthogonal, may impose steric or electronic hindrance that interferes with nearby imine bonding. Overall, achieving optimal material performance requires balancing competition and synergy among these three dynamic bonding mechanisms to direct network architecture and responsiveness.

The ionic radius regulation strategy can be extended to other ionic liquid-based hydrogel systems. When ionic liquids are incorporated into hydrogel networks, the interplay of ionic radii becomes noticeable in affecting the conductivity, mechanical strength, and swelling behaviors of the network. Smaller ions with higher mobility typically led to higher ionic conductivity within the ionic liquid-based hydrogel. For example, Pal and Ghosh studied ionic conduction mechanisms of hydrogels using three different ionic liquids (i.e., BDMIMBF_4_, BMIMBF_4_, and EMIMBF_4_) that had the same anions but different imidazolium cations.^65^ With the smallest size of cations, the hydrogel doped with EMIMBF_4_ showed the highest free ion diffusivity, resulting in the highest ionic conductivity. The mechanical properties of ionic liquid-based hydrogel are also highly dependent on the type of ions present. For example, Jastram et al. developed a hydrogel system based on polymeric ionic liquids, wherein the choice of ionic constituents was tailored according to the charge of the polymer backbone.^66^ For positively charged networks, chloride and bromide anions were employed, while potassium cations were used in hydrogels with negatively charged polymer backbones. The selected ions span a wide range of ionic radii (i.e., 181 pm for chloride, 196 pm for bromide, and 138 pm for potassium), resulting in distinct differences in charge density. Smaller potassium ions exhibited higher charge density and formed stronger electrostatic interactions with the polymer matrix compared to the larger chloride and bromide anions. These intensified ionic interactions contribute to the formation of a denser polymer network, enhancing the mechanical robustness of the hydrogel network.

### 2.3 Volatile Organic Compound Sensing

The PPG/ADA-Ln^3+^ lyophilized hydrogels were applied for volatile organic compound (VOC) sensing. In a customized container, a PPG/ADA-Ln^3+^ lyophilized hydrogel was placed on top of the cut dropper in the vial containing various VOCs (Figure 5a). The luminescence changes of the PPG/ADA-Ln^3+^ lyophilized hydrogels after sensing VOCs were analyzed by an instrument with an integrating sphere and spectrometer at a laser excitation wavelength of 360 nm (Figure 5a). In a time-dependent experiment, the luminescence of the PPG/ADA-Ln^3+^ lyophilized hydrogels decreased in the presence of VOCs (i.e., acetone, ethyl acetate (EA), ethanol (EtOH), formaldehyde (FA), acetic acid (HOAc), and ammonia (NH_3_)), and the changes of the luminescence intensity reached a plateau after 45 min (Figure S11). To ensure that the VOCs were fully saturated in the container, the VOC sensing was conducted by exposing the PPG/ADA-Ln^3+^ lyophilized hydrogels to VOCs for 2 h.

**Figure 5:**
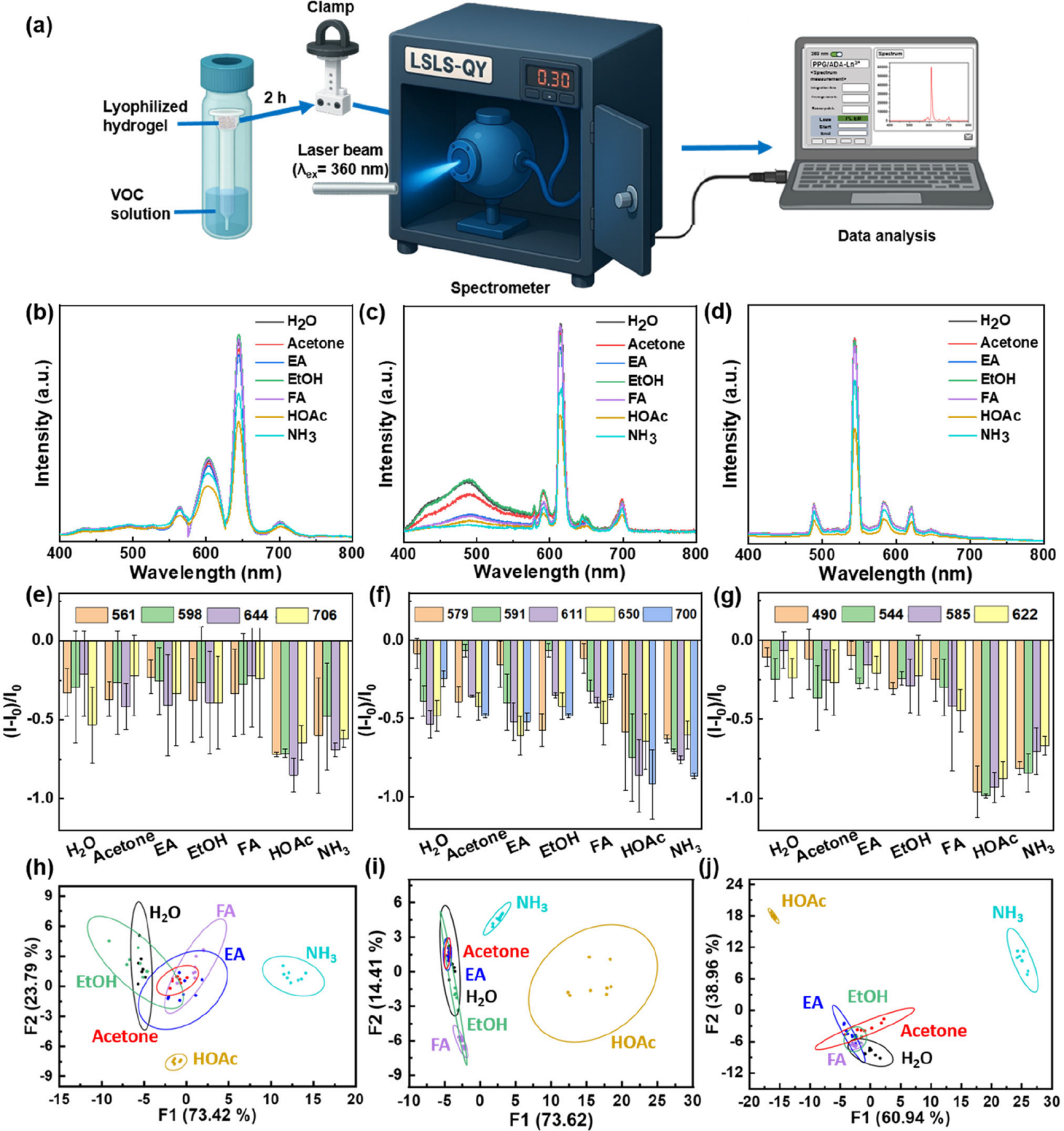
(a) Schematic illustration of using PPG/ADA-Ln^3+^ lyophilized hydrogels for VOC sensing. Luminescence spectra of (b) PPG/ADA-Sm^3+^, (c) PPG/ADA-Eu^3+^, and (d) PPG/ADA-Tb^3+^ lyophilized hydrogels after exposure to VOCs for 2 h. Signal patterns of the luminescence intensity variety ((*I* – *I*_0_)/*I*_0_) of (e) PPG/ADA-Sm^3+^, (f) PPG/ADA-Eu^3+^, and (g) PPG/ADA-Tb^3+^ lyophilized hydrogels for sensing different VOCs. Score plot for VOC sensing of (h) PPG/ADA-Sm^3+^, (i) PPG/ADA-Eu^3+^, and (j) PPG/ADA-Tb^3+^ lyophilized hydrogels obtained from LDA (*n* = 8).

The luminescence spectra of the PPG/ADA-Ln^3+^ lyophilized hydrogels after exposure to VOCs were analyzed through the characteristic luminescence peaks of lanthanides. The ratio of *I* – *I*_0_ to *I*_0_ in the spectra of lyophilized hydrogels was employed for quantitative comparison, where *I*_0_ and *I* denote the luminescence intensity before and after sensing, respectively. The results showed that the luminescence of the PPG/ADA-Ln^3+^ lyophilized hydrogels was quenched after exposure to VOCs, with HOAc and NH_3_ causing a more notable quenching effect compared to other VOCs (Figure 5b–d). Also, unique luminescence quenching patterns for each VOC were obtained after the analysis based on the luminescence changes (Figure 5e–g). These luminescence quenching patterns were further processed using linear discriminant analysis (LDA), showing that HOAc and NH_3_ can be differentiated from the other tested VOCs (Figure 5h–j). In particular, the sum of the canonical variable values for the LDA of PPG/ADA-Tb^3+^ lyophilized hydrogels (99.90%) was higher than that for PPG/ADA-Sm^3+^ (97.21%) and PPG/ADA-Eu^3+^ (88.03%) lyophilized hydrogels, indicating PPG/ADA-Tb^3+^ lyophilized hydrogel exhibited stronger class discriminability than PPG/ADA-Sm^3+^ and PPG/ADA-Eu^3+^ lyophilized hydrogels.

The substantial luminescence quenching of the PPG/ADA-Ln^3+^ lyophilized hydrogels induced by HOAc and NH_3_ vapors is illustrated in Scheme 3. Specifically, HOAc vapor can alter the environmental pH in the presence of water, where the resulting acidic conditions protonate the carboxylate groups to weaken or disrupt their coordination with Ln^3+^ ions within the hydrogel network.^67^ On the other hand, NH_3_ vapor, also in the presence of water, creates a basic environment that facilitates the formation of hydroxides such as Ln­(OH)_3_.^68^ As a result, the acidic and basic conditions generated by HOAc and NH_3_ vapors, respectively, lead to pronounced luminescence quenching of the PPG/ADA-Ln^3+^ lyophilized hydrogels, surpassing the effects observed with other VOCs.

**Scheme 3:**
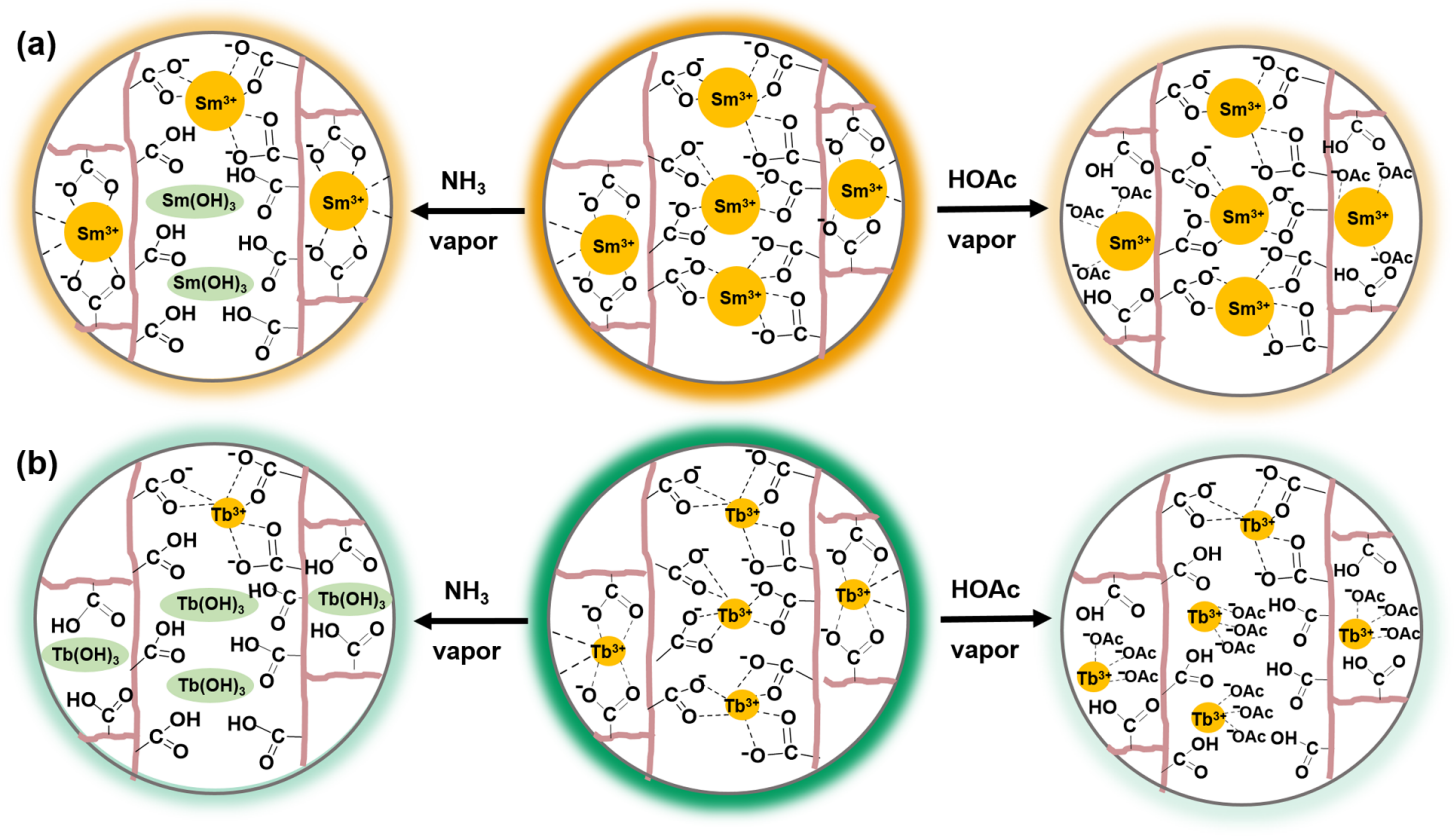
Schematic Illustrations of the Luminescence Quenching Mechanisms of (a) PPG/ADA-Sm^3+^ and (b) PPG/ADA-Tb^3+^ Lyophilized Hydrogel Networks

The greater quenching efficiency induced by HOAc and NH_3_ in PPG/ADA-Tb^3+^ lyophilized hydrogels, compared to PPG/ADA-Sm^3+^ and PPG/ADA-Eu^3+^ lyophilized hydrogels, can be attributed to differences in the ionic radii of the lanthanides. In an acidic environment, HOAc may displace carboxylate groups from a weakened Ln^3+^-carboxylate complex. The smaller ionic radius of the Tb^3+^ ion results in stronger interactions between its electrons and surrounding matrix molecules compared to Sm^3+^ and Eu^3+^ ions.^23^ Consequently, HOAc exhibits a higher binding affinity for Tb^3+^ ions than for Sm^3+^ or Eu^3+^ ions, further destabilizing the Ln^3+^-carboxylate complex and significantly disrupting its coordination. In the NH_3_ vapor-induced basic environment, the higher charge density of the smaller Tb^3+^ ion enhances its electrostatic attraction to hydroxide ions (OH^–^), promoting the formation of Tb­(OH)_3_ over Sm­(OH)_3_ and Eu­(OH)_3_.^48^ Consequently, NH_3_ vapor more effectively induced Tb­(OH)_3_ formation, leading to a more pronounced reduction in the luminescence of the Tb^3+^-carboxylate network.

Fourier transform infrared (FTIR) spectroscopy was employed to analyze changes in the chemical structures of lyophilized hydrogels following sensing. After HOAc vapor sensing, the carbonyl (C=O) stretching vibration shifted from 1640 to 1657 cm^–1^ in the FTIR spectrum of PPG/ADA-Sm^3+^ lyophilized hydrogel (Figure S12a), while the band shifted from 1643 to 1688 cm^–1^ for PPG/ADA-Tb^3+^ lyophilized hydrogel (Figure S12b). Given the characteristic FTIR frequencies of carboxylic acids and carboxylates, the observed shift in the C=O stretching vibration could be due to the protonation of the carboxylate groups within the network by HOAc vapor. The more pronounced shift observed in the PPG/ADA-Tb^3+^ lyophilized hydrogel compared to the PPG/ADA-Sm^3+^ lyophilized hydrogel suggested that a greater amount of carboxylic acid was generated in the former after sensing HOAc vapor, as shown in Scheme 3b. On the other hand, after sensing NH_3_ vapor, asymmetric stretching vibration of a carboxylate (COO^–^) group was observed at 1576 cm^–1^ in the FTIR spectrum of PPG/ADA-Tb^3+^ lyophilized hydrogel, which was more apparent than that in the FTIR spectrum of PPG/ADA-Sm^3+^ lyophilized hydrogel. The results revealed that a higher quantity of COO^–^ groups was produced in the PPG/ADA-Tb^3+^ lyophilized hydrogel upon exposure to NH_3_ vapor compared to the PPG/ADA-Sm^3+^ lyophilized hydrogel, as shown in Scheme 3b.

The limit of detection (LOD) of the three types of PPG/ADA-Ln^3+^ hydrogels in sensing HOAc was further determined to compare their sensing capabilities. The PPG/ADA-Ln^3+^ hydrogel was exposed to HOAc solutions with varying concentrations to obtain the relationship between the luminescence change and the HOAc vapor concentration (Figure S13). The LOD of the PPG/ADA-Ln^3+^ hydrogel was calculated by eq 8 in the experimental section (see Supporting Information). The LOD of HOAc for PPG/ADA-Sm^3+^, PPG/ADA-Eu^3+^, and PPG/ADA-Tb^3+^ lyophilized hydrogels was 0.406, 0.325, and 0.128 ppm, respectively, which were much lower than the World Health Organization (WHO) standard (10 ppm) for HOAc vapor.^69^ The lower LOD of PPG/ADA-Tb^3+^ hydrogel compared to PPG/ADA-Sm^3+^ and PPG/ADA-Eu^3+^ lyophilized hydrogels could be the result of a stronger electrostatic force between extra-nuclear electrons with a smaller radius of Tb^3+^ ions and matrix molecules.^23^

Detecting gaseous acid is essential as the acidic vapor is corrosive to skin, and inhalation injury caused by acidic vapor may lead to damage to the bronchus and pulmonary edema. Luminescent lanthanide-containing materials (e.g., MOFs,^70,71^ filter paper with Ln-containing complexes,^72^ carbon dot-based nanocomposites,^73^ and aminoclays^74^) have been demonstrated for gaseous acid detection (Table 1). In this study, the PPG/ADA-Ln^3+^ lyophilized hydrogel has shown the potential to distinguish the HOAc vapor among the VOCs through LDA, with a low LOD of 0.128 ppm using PPG/ADA-Tb^3+^ lyophilized hydrogel. Compared to current powder-based sensing systems, PPG/ADA-Ln^3+^ lyophilized hydrogel-based sensors offer several advantages for acidic vapor sensing. The robust porous structure of lyophilized hydrogel ensures mechanical stability, preventing aggregation or settling, while enhancing reusability through simplified regeneration. Their porous structure also provides a large surface area, potentially enabling faster vapor diffusion and response times compared to the limited porosity and slower analyte diffusion in powder beds. In particular, lyophilized hydrogel could be easily molded into specific shapes, handled, and integrated into sensor platforms or wearable devices compared to powders. These features (i.e., improved stability, porous structure, easy handling, and great flexibility in design and application) position lyophilized hydrogels as a versatile platform for high-performance acidic vapor detection.

**Table 1:** Lanthanide-Containing Materials Are Used for Acidic Vapor Detection

materials	lanthanide ions	target	LOD	ref
3D Ln-MOFs^a^	Pr^3+^, Ce^3+^	HCl vapor	0.18 ppm	^70^
Ln-MOF^b^	Eu^3+^, Tb^3+^, and Gd^3+^	picric acid vapor	N.A.	^71^
Ln-PBA^c^	Eu^3+^, Tb^3+^	HCl vapor	N.A.	^72^
CDs-Ln-HL^d^ ^,^ ^e^	Eu^3+^	HCl vapor	N.A.	^73^
AC-Ln(DPA)_*n*_ ^f^ ^,^ ^g^	Eu^3+^, Tb^3+^	HCl vapor	N.A.	^74^
PPG/ADA-Ln^3+^	Sm^3+^, Eu^3+^, and Tb^3+^	acetic acid vapor	0.128 ppm	this work

a MOFs = H_3_O·[Ln_3_(TBAPy)_2_(μ_2_-H_2_O)_2_(OH)_2_]·2DMA·2diox·6.5H_2_O, where TBAPy = (1,3,6,8-tetrakis­(*p*-benzoic acid)­pyrene), DMA = *N*,*N*-dimethylacetamide, and Diox = 1,4-dioxane.

b MOF = methyl 4-(chlorocarbonyl)­benzoate, methyl 3,4-diaminobenzoate.

c PBA = *N*-(2-pyridinyl)­benzoylacetamide.

d CDs = fluorescent carbon dots.

e HL = amide-type β-diketone ligand *N*-(2-pyridinyl)­benzoylacetamide.

f AC = aminoclay.

g DPA = pyridine-2,6-dicarboxylic acid.

The VOC sensing results showed that PPG/ADA-Ln^3+^ hydrogels can distinguish acidic vapors under LDA. Given that the acidic vapors could be one of the gaseous products generated by bacteria (i.e., Lactic acid bacteria,^75^ *Pseudomonas oleovorans*,^76^ and *Staphylococcus aureus* (*S. aureus*)^77^), the PPG/ADA-Ln^3+^ lyophilized hydrogels were further applied for bacteria sensing (Figure S14a). The characteristic luminescence of the three PPG/ADA-Ln^3+^ lyophilized hydrogels exposed to *S. aureus* were more noticeably decreased compared to the exposure to the tryptic soy broth (TSB) control group as well as to *Escherichia coli* (*E. coli*) and *Salmonella enterica* (*S. enterica*) (Figures 6a–f and S14b–d), further resulting in *S. aureus* can be distinguished from the tested groups under LDA (Figure 6g–i). These results could be due to the acid-induced changes in the luminescence of lyophilized hydrogels, as 3-methyl-butanoic acid was a unique volatile biomarker from the growth of *S. aureus* compared to that of *E. coli* and *S. enterica* through the analysis of gas chromatography-mass spectroscopy.^77^

**Figure 6:**
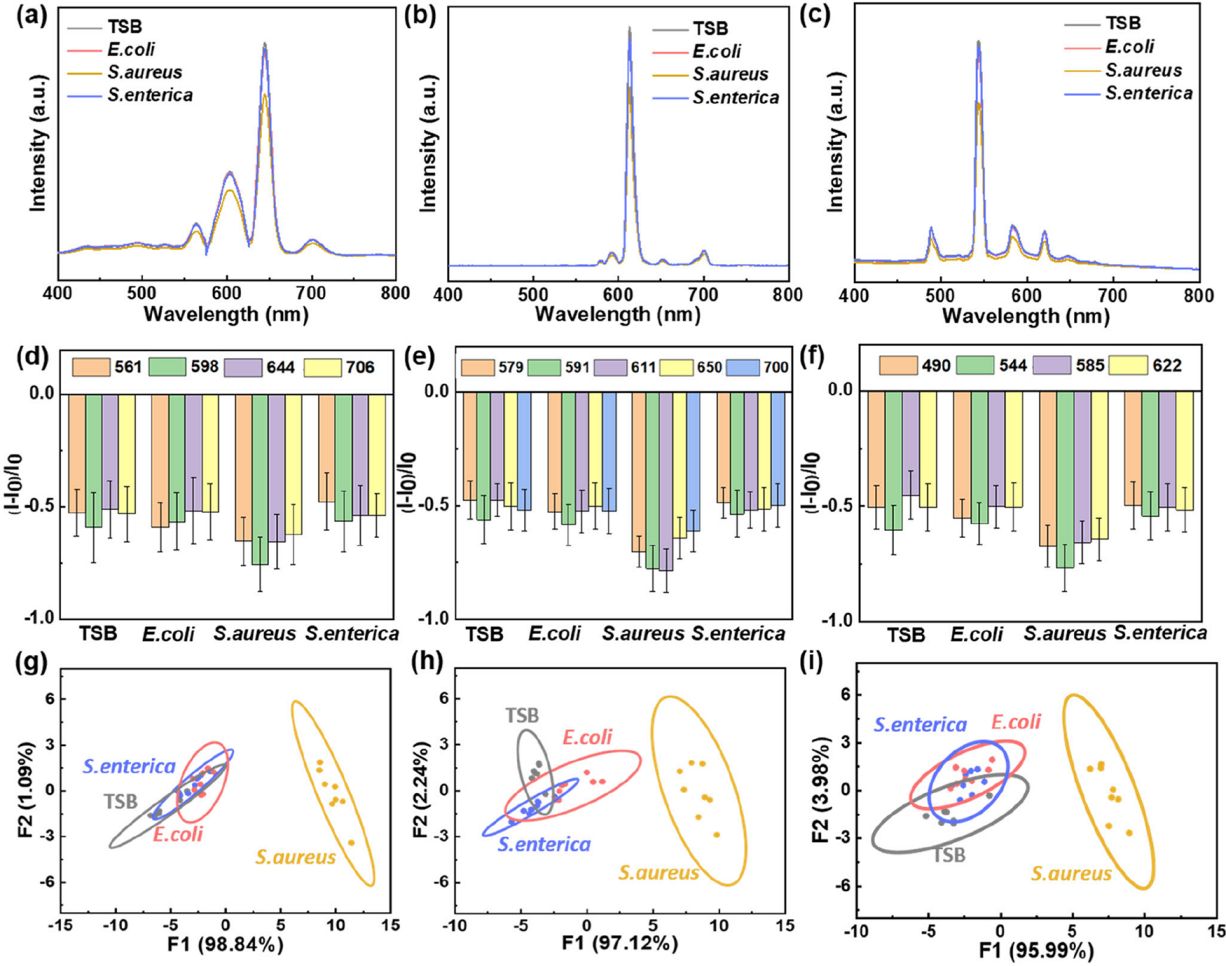
PPG/ADA-Ln^3+^ lyophilized hydrogels used for bacteria sensing. Luminescence spectra of (a) PPG/ADA-Sm^3+^, (b) PPG/ADA-Eu^3+^, and (c) PPG/ADA-Tb^3+^ lyophilized hydrogels after exposure to bacteria for 2 h. Signal patterns of the luminescence intensity variety ((*I* – *I*_0_)/*I*_0_) of (d) PPG/ADA-Sm^3+^, (e) PPG/ADA-Eu^3+^, and (f) PPG/ADA-Tb^3+^ lyophilized hydrogels for sensing different bacteria. Score plot for bacteria sensing of (g) PPG/ADA-Sm^3+^, (h) PPG/ADA-Eu^3+^, and (i) PPG/ADA-Tb^3+^ lyophilized hydrogels obtained from LDA (*n* = 8).

The luminescence response of the PPG/ADA-Ln^3^^+^ lyophilized hydrogel to *S. aureus* presented a time-dependent manner, showing the luminescence intensity of the lyophilized hydrogels stabilized and reached a plateau after 2 h of exposure (Figure S15). The LOD of the three types of PPG/ADA-Ln^3+^ lyophilized hydrogels in sensing *S. aureus* was further determined to compare their sensing capabilities. The PPG/ADA-Ln^3+^ hydrogel was exposed to *S. aureus* with varying concentrations (Figure S16), and the LOD of *S. aureus* for PPG/ADA-Sm^3+^, PPG/ADA-Eu^3+^, and PPG/ADA-Tb^3+^ lyophilized hydrogels were about 645,000, 484,000, and 323,000 CFU/mL, respectively. Similar to the HOAc vapor sensing results, PPG/ADA-Tb^3+^ lyophilized hydrogel presented the lowest LOD in sensing *S. aureus* compared to PPG/ADA-Sm^3+^ and PPG/ADA-Eu^3+^ lyophilized hydrogels. These results should be due to a better binding affinity and stronger interaction between the small-sized Tb^3+^ ions and the surrounding matrix. This suggests that Tb^3+^ ions, having the smallest ionic radius among the three tested lanthanide ions, would exhibit the strongest interaction with the carboxylate group of acid vapor (e.g., 3-methyl-butanoic acid) produced during the *S. aureus* growth due to a higher charge density and thus stronger electrostatic attraction.

Current lanthanide-containing hydrogel systems used for bacteria sensing focus on monitoring the bacterial growth by showing the luminescence changes.^30,31^ Here, we demonstrated that the PPG/ADA-Ln^3+^ lyophilized hydrogels were capable of differentiating bacteria based on the gaseous VOCs generated from the growth of bacteria. PPG/ADA-Ln^3+^ hydrogel can distinguish *S. aureus* from *E. coli* and *S. enterica* through LDA, with PPG/ADA-Tb^3+^ lyophilized hydrogel presenting the lowest LOD of 323,000 CFU/mL in detecting *S. aureus*. Given that *S. aureus* has been proven to be related to some diseases (e.g., worsened eczema and asthma symptoms),^78^ the ability to distinguish *S. aureus* makes PPG/ADA-Ln^3+^ lyophilized hydrogels a promising platform for practical applications. For example, in clinical diagnostics, accurately distinguishing *S. aureus* from other bacterial pathogens ensures appropriate antibiotic prescriptions, reducing the risk of antimicrobial resistance. Similarly, in food safety, differentiating *S. aureus* from other contaminants in food is crucial for accurate hazard assessments and effective mitigation strategies.

## 4 Conclusions

A series of triple-cross-linked luminescent PPG/ADA-Ln^3+^ hydrogels have been successfully fabricated by incorporating three lanthanide ions with varying ionic radii (i.e., Sm^3+^, Eu^3+^, or Tb^3+^) to understand the structure–property–function relationships of the hydrogels. The study revealed that PPG/ADA-Tb^3+^ hydrogels exhibited the densest network, highest mechanical strength, minimal swelling behavior, superior stability, and the longest self-healing time compared to PPG/ADA-Sm^3+^ and PPG/ADA-Eu^3^^+^ hydrogels. PPG/ADA-Ln^3+^ hydrogels also demonstrated promising differentiation capabilities for VOCs and bacteria. Lyophilized PPG/ADA-Ln^3+^ hydrogels were able to distinguish HOAc and NH_3_ from other VOCs through LDA. Furthermore, we extended this capability to the challenging task of bacterial differentiation, successfully distinguishing *S. aureus* from *E. coli* and *S. enterica* based on the unique acidic volatile biomarkers produced during *S. aureus* growth. Notably, PPG/ADA-Tb^3+^ lyophilized hydrogel presented the lowest LOD among the three PPG/ADA-Ln^3+^ hydrogels in detecting VOCs and *S. aureus*. Overall, the superior structural, mechanical, and sensing properties of PPG/ADA-Tb^3+^ hydrogel should be attributed to the smaller ionic radius of Tb^3+^ ions, which allows for optimized coordination with the carboxylate groups of the polymers. Taken together, our study provides a comprehensive understanding of how lanthanide ionic radii dictate the structural and functional characteristics of these triple-cross-linked luminescent hydrogels. This finding provides crucial insights into the structure–property-function relationships in lanthanide-containing hydrogels, paving the way for the rational design of materials with tailored mechanical and sensing performance. The demonstrated versatility and exceptional sensing capabilities position these materials as promising candidates for a wide range of applications in diverse fields such as environmental monitoring, clinical diagnostics, and food safety.

## Supplementary Material



## Notes

Materials and methods; NMR spectrum of ADA; XPS and FTIR spectra of lyophilized hydrogels; luminescence spectra of hydrogels and lyophilized hydrogels; rheology, tensile testing, and self-healing efficiency data of hydrogels; images of the setup and luminescence change for bacteria sensing; and XPS, micro-CT, and rheology analyses (PDF)
