# Supplementary material for: Lanthanide Ionic Radius Modulation for Tailored Triple-Cross-Linked Luminescent Gelatin/Alginate Hydrogels: Structural, Mechanical, and Sensing Insights
Source: JACS Au. 2025 Aug 13;5(8):4022–35. doi: 10.1021/jacsau.5c00647 (PMC12381702; doi:10.1021/jacsau.5c00647)
Supplement: Supplementary file 1 [file au5c00647_si_001.pdf]

Supporting information

Lanthanide Ionic Radius Modulation for Tailored Triple-Crosslinked Luminescent Gelatin/Alginate Hydrogels: Structural, Mechanical, and Sensing Insights

Shu-Ying Wu[a], Yu-Ning An[a], and Yi-Cheun Yeh[a*]

[a] Institute of Polymer Science and Engineering, National Taiwan University, Taipei 10617, Taiwan

* Corresponding author.

E-mail address: yicheun@ntu.edu.tw

Contents

1. Materials and methods

2. Figures S1 to S14

3. Table S1 to S3

4. References

1. Materials and methods

Materials

Gelatin (from bovine skin, Type B) and 1-ethyl-3-(3-dimethylaminopropyl) carbodiimide hydrochloride (99%) (EDC) were purchased from Sigma-Aldrich. 4-Formylphenylboronic acid, sodium cyanoborohydride, sodium meta periodate, diethylene glycol, and N-hydroxysuccinimide (98%) (NHS) were purchased from Alfa Aesar. Sodium alginate and 2-(N-morpholino)ethanesulfonic acid (MES) (>99%, <1% water) were purchased from ACROS. Samarium chloride hexahydrate ($SmCl_3•(H_2O)_6$, 99.9%), europium(III) chloride hexahydrate ($EuCl_3•(H_2O)_6$, 99.9%), and terbium chloride hexahydrate ($TbCl_3•(H_2O)_6$, 99.9%) were purchased from MorrChem. Acetic acid (HOAc, >99.8%) was purchased from Fluka™. Ammonium hydroxide (NH_4OH, 28%) was purchased from CMOS. Ethyl Acetate (EA, >99.5%) was purchased from Seedchem. Ethanol (EtOH, 95%) was purchased from Taiwan sugar corporation. Acetone was purchased from Everdine Technology. Tryptic Soy Broth (TSB) was purchased from HiMedia Laboratories Pvt. Ltd. Agar powder (bacteriological grade) was obtained from Bioshop Canada Inc. Bacterial strains *Escherichia coli* (*E. coli*) (BCRC 13055), *Staphyloccus aureus* (*S. aureus*) (BCRC 13077), and *Salmonella enterica* (*S. enterica*) subsp. *enterica* (BCRC 12947) were purchased from the Bioresource Collection and Research Center in Taiwan.

Characterization techniques

The hydrogels were freeze-dried using a lyophilizer (UNISS FDM-2). The pore size of the hydrogels was examined by scanning electron microscopy (SEM, TM3000, FlexSEM1000 II, Hitachi Ltd., Japan). Fourier-transform infrared (FT-IR) spectra were obtained with Spectrum Two (PerkinElmer). The molecular weight of polymers was measured by gel permeation chromatography (GPC) and conducted on an Enshine SUPER CO-150 system equipped with an RI-2031 detector using HPLC-grade water as the eluent. Image J (National Institutes of Health) was used to determine the pore size distribution. The average pore size of each hydrogel was calculated based on 30 different pores, and each pore was measured 30 times from different directions. Micro-CT (Skyscan 1076) was employed to investigate the internal porous structure of the hydrogels. The element compositions and chemical state of hydrogels were analyzed using X-

ray photoelectron spectroscopy (XPS, ULVAC PHI 5000 Versa Probe, Japan). Rheological analyses of hydrogels were carried out using a rheometer (AR2000EX, TA Instrument) with an 8 mm steel parallel plate. The luminescence and photoluminescence quantum yield of the hydrogels were analyzed by a photoluminescence and efficiency measurement system (LSLS-QY, LiveStrong Optoelectronics) with an integrating sphere and an excitation at 360 nm. Compression tests were performed using a mechanical testing machine (AGS-500N, Shimadzu) with a 10 N load cell.

Preparation of PPG/ADA-Ln^{3+} hydrogels

PPG and ADA were synthesized based on our previous work.[1] For hydrogel formation, PPG (15 wt%) and ADA (10 wt%) solutions were prepared in DI water, and the lanthanide ions (i.e., Sm^{3+}, Eu^{3+}, or Tb^{3+}) were added to the PPG solution with a final concentration of 0.002 mole/ml. The two solutions were mixed with equal volume through a dual-channel syringe and left at 25℃ overnight to ensure PPG/ADA-Ln^{3+} hydrogel formation. PPG/ADA-Ln^{3+} hydrogels were put into a refrigerator (-80 °C) for 24 hrs and then lyophilized before sensing application.

Rheological and mechanical measurements of hydrogels

For rheological tests, the hydrogels were placed on the plate of the rheometer. During oscillation strain sweeps, the strain ranged from 0.01% to 1000%, maintained at 1 Hz at 25°C, and data were collected at 10 points per decade. The stress relaxation test was carried out by rheological measurements, under 1% strain, 25°C, and the duration was 120 seconds.

The compressive modulus of the hydrogels was determined by a mechanical testing machine equipped with a 10 N load cell and operated at a constant rate of 1 mm/min. Prior to testing, the cylinder-shaped hydrogels (diameter= 4.5 mm, height= 4.5 mm) were measured precisely using a vernier caliper. The compressive modulus was calculated from the slope of the linear region of the stress-strain curve within the strain range of 10% to 20%.

Tensile testing was conducted using the instrument of the materials testing system equipped with a 10 N load cell. For tensile testing, hydrogels were formed into dog-bone-shaped

polydimethylsiloxane (PDMS) molds (2.6 mm thick, 5.0 mm wide at center). Force-displacement curves obtained from the machine were converted to stress-strain curves.

Swelling ratio and water content of hydrogels

The lyophilized hydrogels were weighed (w_d) (~ 19 mg), shaken on a digital rotator at 100 rpm, and immersed in DI water (1 mL) at 37 °C. After that, the hydrogels were cautiously taken out of the solution, any leftover DI water was cleaned off the surface of the hydrogels, and re-weighed immediately to determine the weight after swelling (w_s). The following formula Eq. (1) was used to calculate the swelling ratio:

$$\text{swelling ratio} = \frac{w_s}{w_d} \tag{1}$$

The following Eq. (2) was used to determine the water content:

$$\text{water content (\%)} = \frac{w_s - w_d}{w_s} \times 100\% \tag{2}$$

Degradation behaviors of hydrogels

Lyophilized hydrogels (~ 17 mg) were made for the degradation test. The lyophilized hydrogels (M_0) were immersed in distilled water (1 mL) at 25°C. The hydrogels were frozen, lyophilized, and reweighted at different time intervals (1, 3, 5, and 7 days) (M_d). The weight remaining ratio of lyophilized hydrogels was calculated according to the following Eq. (3):

$$\text{mass remaining(\%)} = \frac{M_d}{M_0} \times 100\% \tag{3}$$

Self-healing behaviors of hydrogels

The self-healing capability of the hydrogel was revealed using cyclic time sweep rheological measurements, wherein the strain amplitude was alternated between 1% and 500% at a constant frequency of 1 Hz and a temperature of 25 °C. Each strain condition was applied for 1 minute to assess the recovery of the storage modulus. The self-healing efficiency of hydrogels was quantified

by determining the compressive modulus changes before and after the healing process, where two cut pieces of hydrogel slowly reunited after contact.

VOC and bacteria sensing using lyophilized hydrogels

PPG/ADA-Ln^{3+} lyophilized hydrogels were placed on a cut dropper and then put into a bottle with 2 mL of various VOCs (i.e., acetone, ethyl acetate (EA), ethanol (EtOH), formaldehyde (FA), acetic acid (HOAc), and ammonia (NH_3)) (1 M) to make the environment full of vapors. After 2 hrs of volatilization, the lyophilized hydrogels were taken out to analyze the luminescence spectra using the LSLS-QY instrument. Data were processed using calculation methods (i.e., $(I-I_0)/I_0$), and the processed data were subjected to linear discriminant analysis (LDA) using XLSTAT software.

To determine the limit of detection (LOD) of HOAc vapor, the lyophilized hydrogels (diameter=5 mm and height=5 mm) were placed in 7 mL vials and then exposed to four different concentrations of HOAc solution (10^{-1}, 10^{-2}, 10^{-3}, and 10^{-4} M; 2 mL) for 2 h. The HOAc vapor concentrations of the solution were calculated by the ideal gas law:[2]

$$P_{HOAc} = \chi_{HOAc} \times Saturation\ vapor\ pressure \tag{4}$$

$$C_{HOAc} = \frac{P_{HOAc}}{RT} \tag{5}$$

Where P_{HOAc} is the partial pressure of HOAc vapor, χHOAc is the mole fraction of HOAc vapor, CHOAc is the concentration of HOAc vapor, R is the gas constant (0.082 L atm K^{-1} mol^{-1}), and T represents the temperature in 298.15 K.

The LOD of the PPG/ADA-Ln^{3+} hydrogel was calculated by the following Eq. (6), (7), and (8):[3]

$$\frac{I_0}{I} = K_{SV}[Q] + 1 \tag{6}$$

$$\sigma = \frac{F_{se}}{F_0} \tag{7}$$

$$LOD = \frac{3\sigma}{K_{sv}} \tag{8}$$

Where I_0 and I were the luminescent intensity before and after sensing, K_{sv} was the Stern–Volmer constant, and [Q] was the quencher concentration (ppm). F_{SE} was the standard error of the fluorescence intensity of the blank sample, and F_o was the luminescence intensity of PPG/ADA-Ln^{3+} hydrogel.[3]

For bacteria sensing, PPG/ADA-Ln^{3+} lyophilized hydrogels were placed on a cut dropper and then put into a tube with 15 mL of tryptic soy broth (TSB) or TSB containing bacteria (i.e., *S. aureus, E. coli,* or *S. enterica* (1.5×10^8 CFU/ml)). After 2 hrs, the lyophilized hydrogels were taken out to analyze the luminescence spectra using the LSLS-QY instrument, and the data were further processed through LDA.

Statistical analysis

Experiments were conducted in triplicate unless otherwise specified in the figure captions. Error bars in the figures represent the standard deviation (s.d.) unless otherwise specified. One-way ANOVA was employed to assess the statistical significance of the differences observed in the data. Significance levels were established at $p< 0.05$, with *, **, and *** denoting p-values of < 0.05, < 0.01, and < 0.001, respectively.

2. Figures S1 to S14

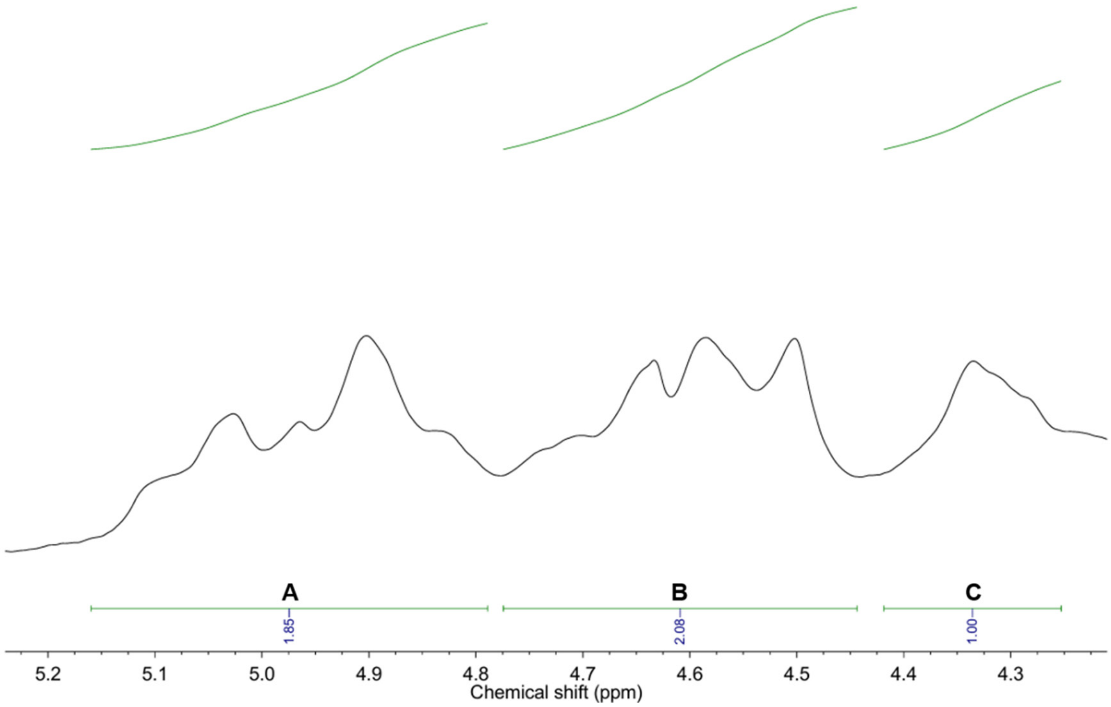

Figure S1. ^{1}H-NMR spectrum of ADA.

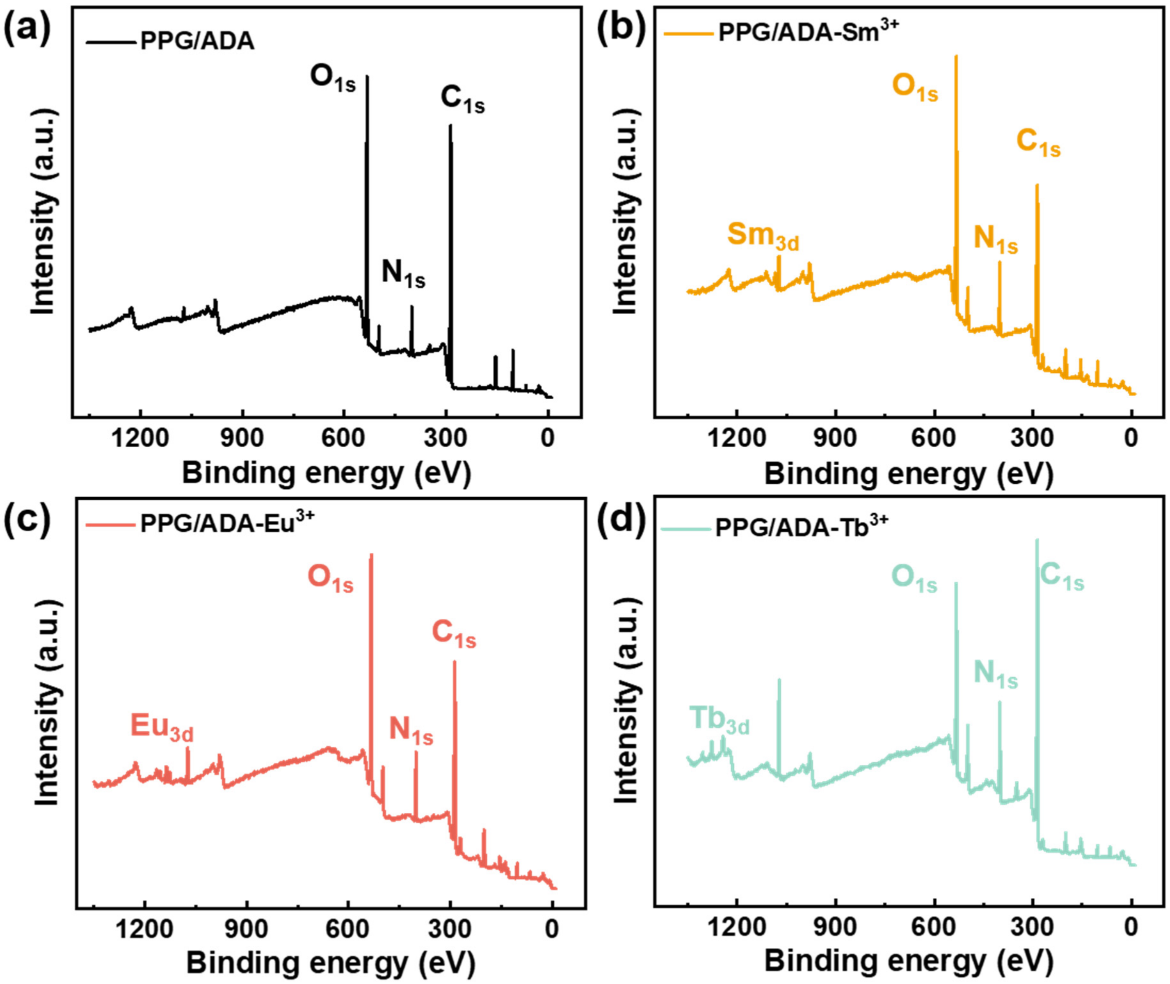

Figure S2. XPS survey spectra of (a) PPG/ADA, (b) PPG/ADA-Sm^{3+}, (c) PPG/ADA-Eu^{3+}, and (d) PPG/ADA-Tb^{3+} lyophilized hydrogels.

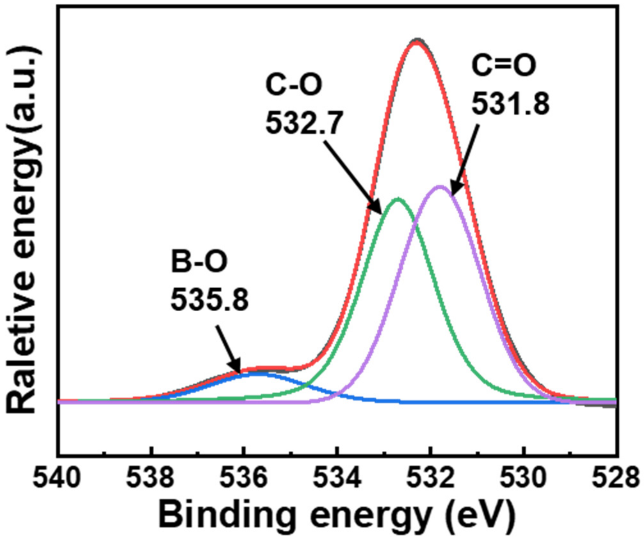

Figure S3. O1s deconvoluted XPS spectrum of PPG/ADA lyophilized hydrogel.

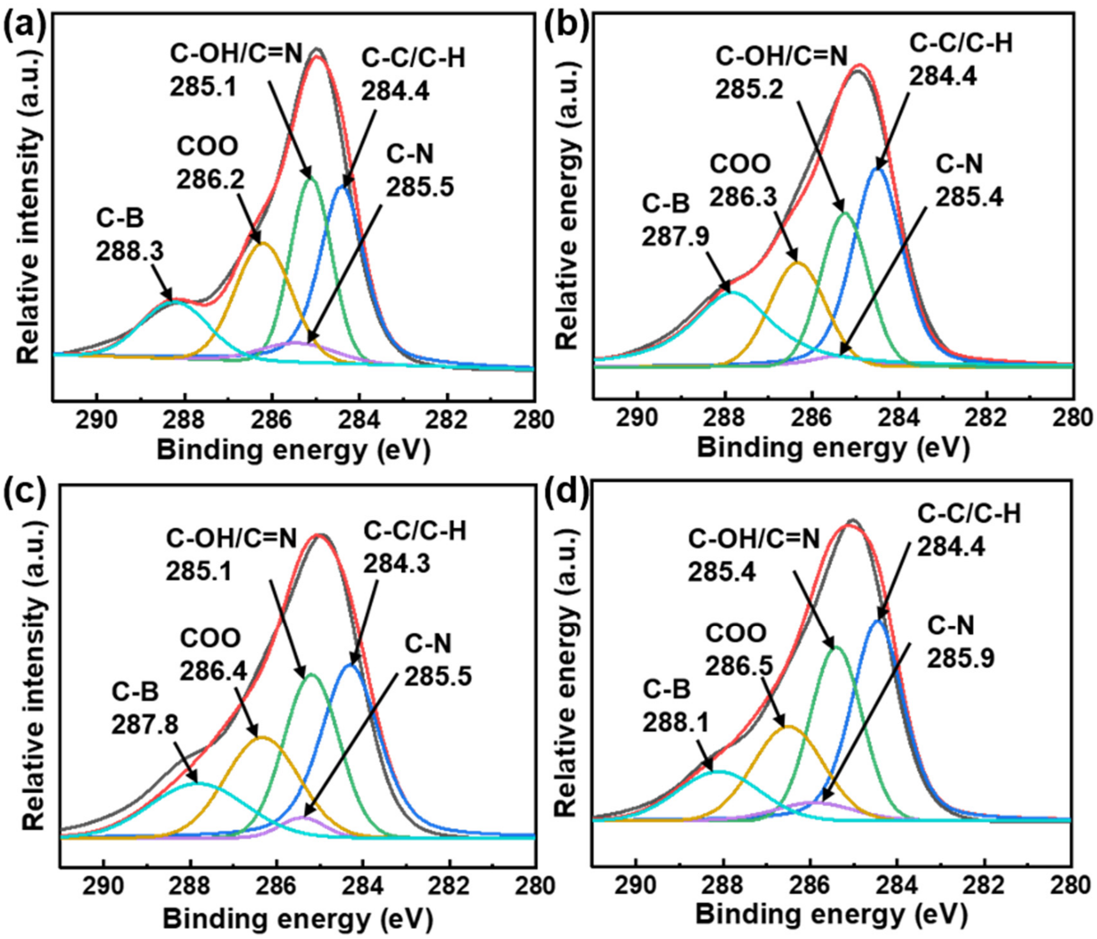

Figure S4. C1s deconvoluted XPS spectra of (a) PPG/ADA, (b) PPG/ADA-Sm^{3+}, (c) PPG/ADA-Eu^{3+}, and (d) PPG/ADA-Tb^{3+} lyophilized hydrogels.

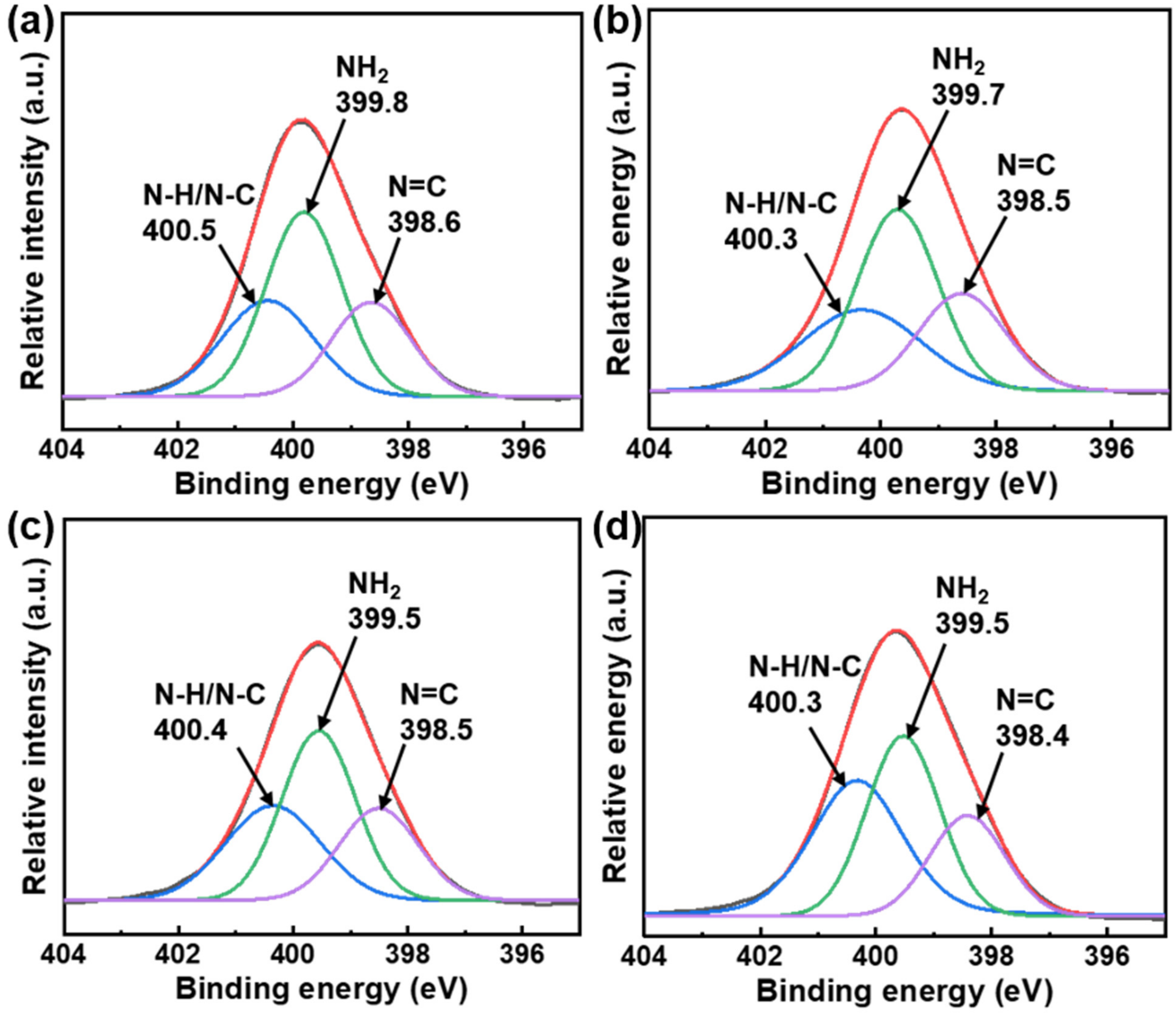

Figure S5. N1s deconvoluted XPS spectra of (a) PPG/ADA, (b) PPG/ADA-Sm^{3+}, (c) PPG/ADA-Eu^{3+}, and (d) PPG/ADA-Tb^{3+} lyophilized hydrogels.

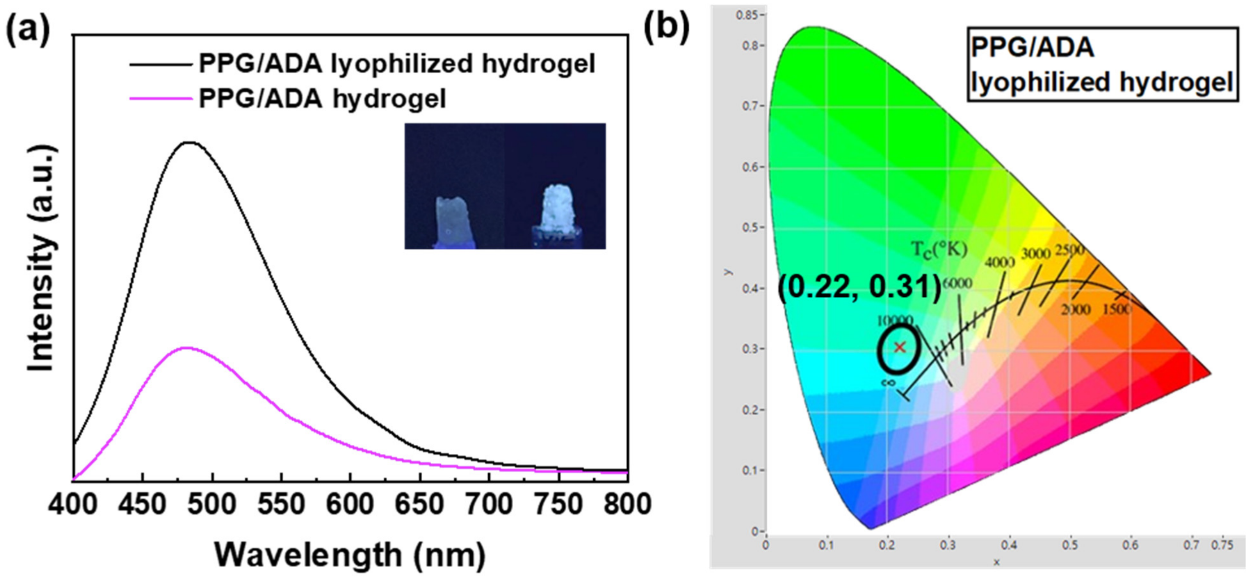

Figure S6. (a) Luminescence spectrum and (b) CIE image of PPG/ADA hydrogel. Inset: images of hydrogel (left) and lyophilized hydrogel (right) under UV light (365 nm).

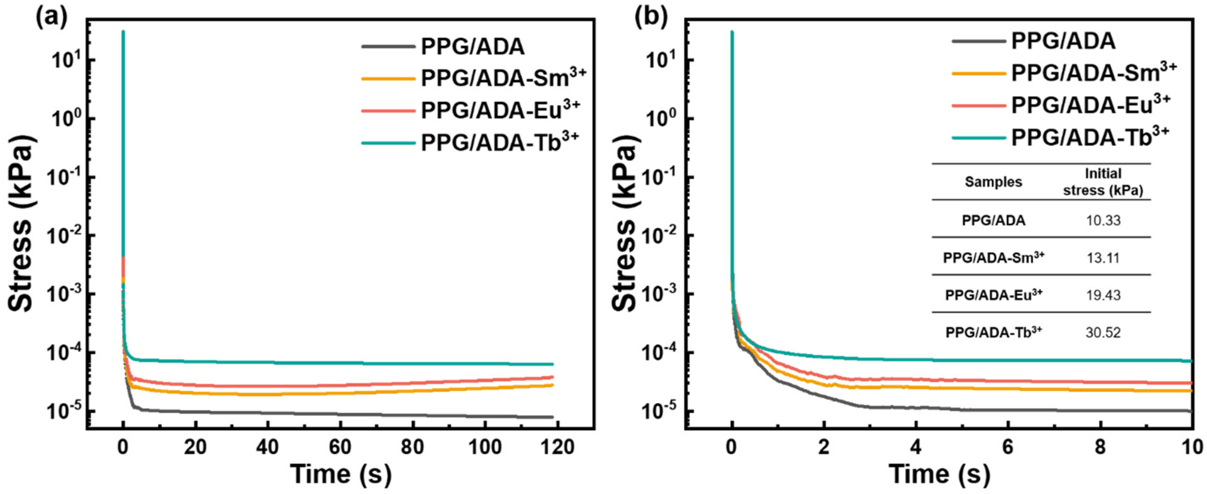

Figure S7. Stress relaxation behaviors of hydrogels under different time periods: (a) 120 and (b) 10 sec.

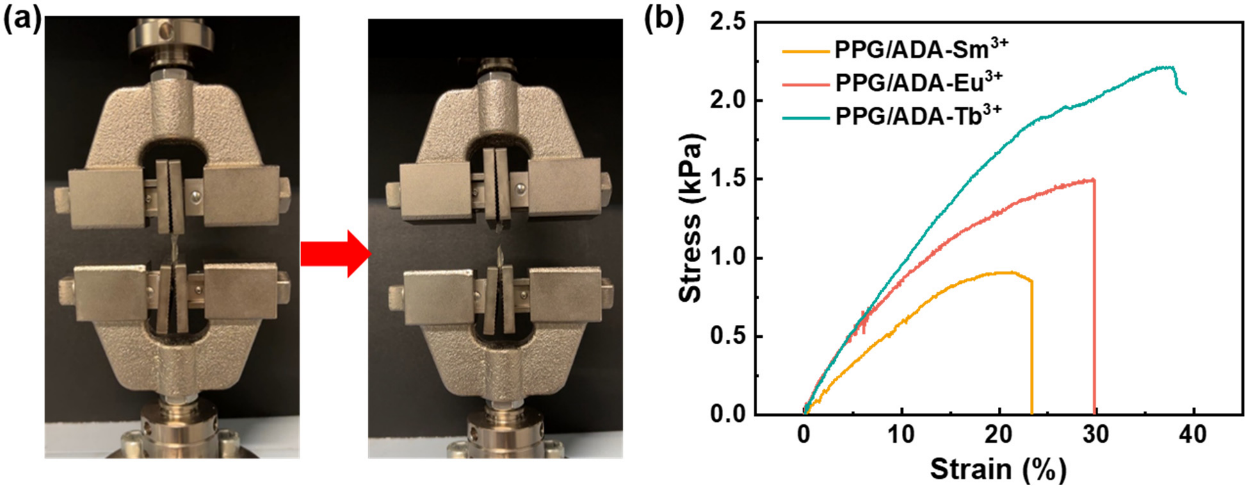

Figure S8. (a) Photographs of the hydrogel before and after tensile loading, with images taken immediately before failure. (b) Tensile mechanical performance of hydrogels.

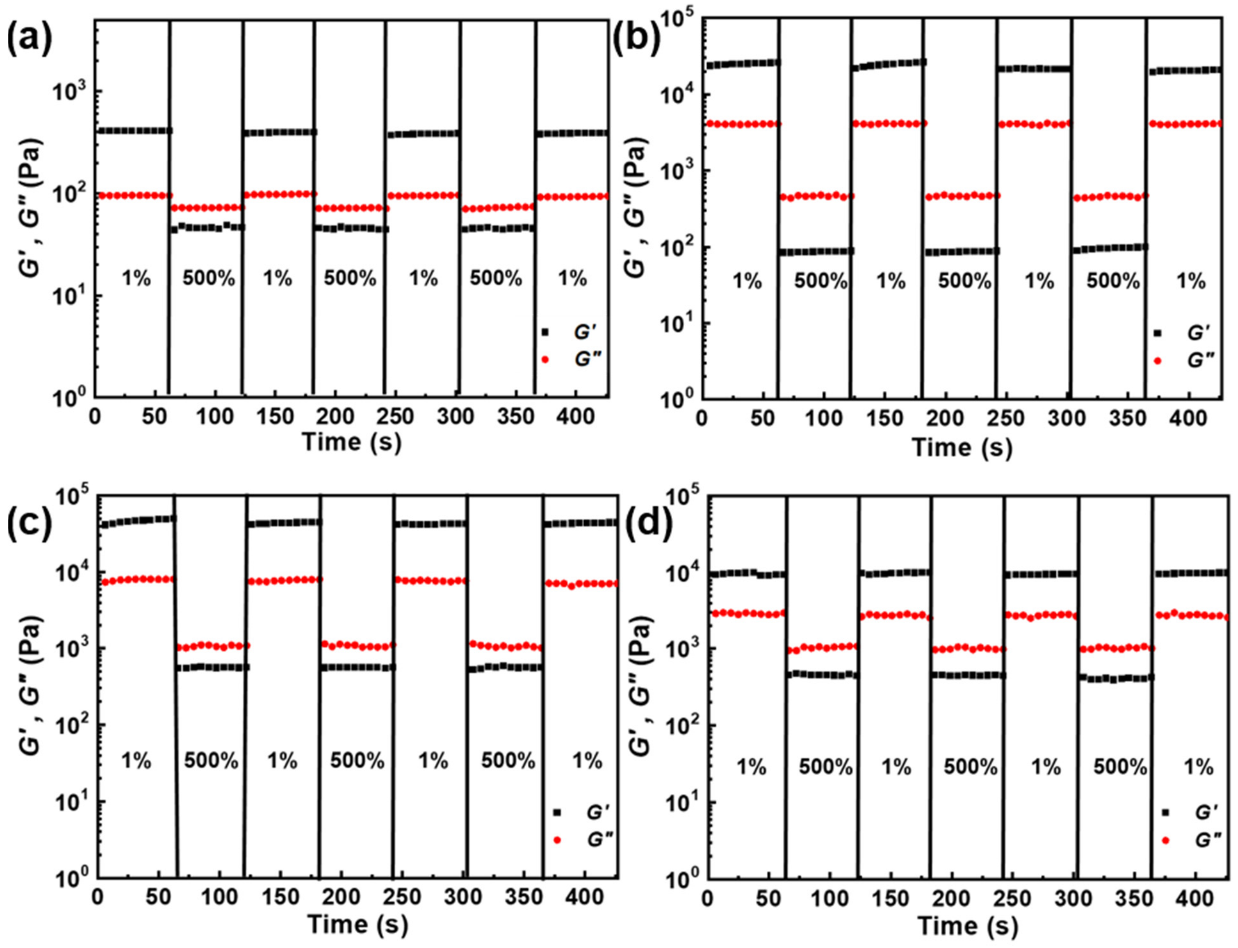

Figure S9. Cyclic strain sweeps of (a) PPG/ADA, (b) PPG/ADA-Sm^{3+}, (c) PPG/ADA-Eu^{3+}, and (d) PPG/ADA-Tb^{3+} hydrogels.

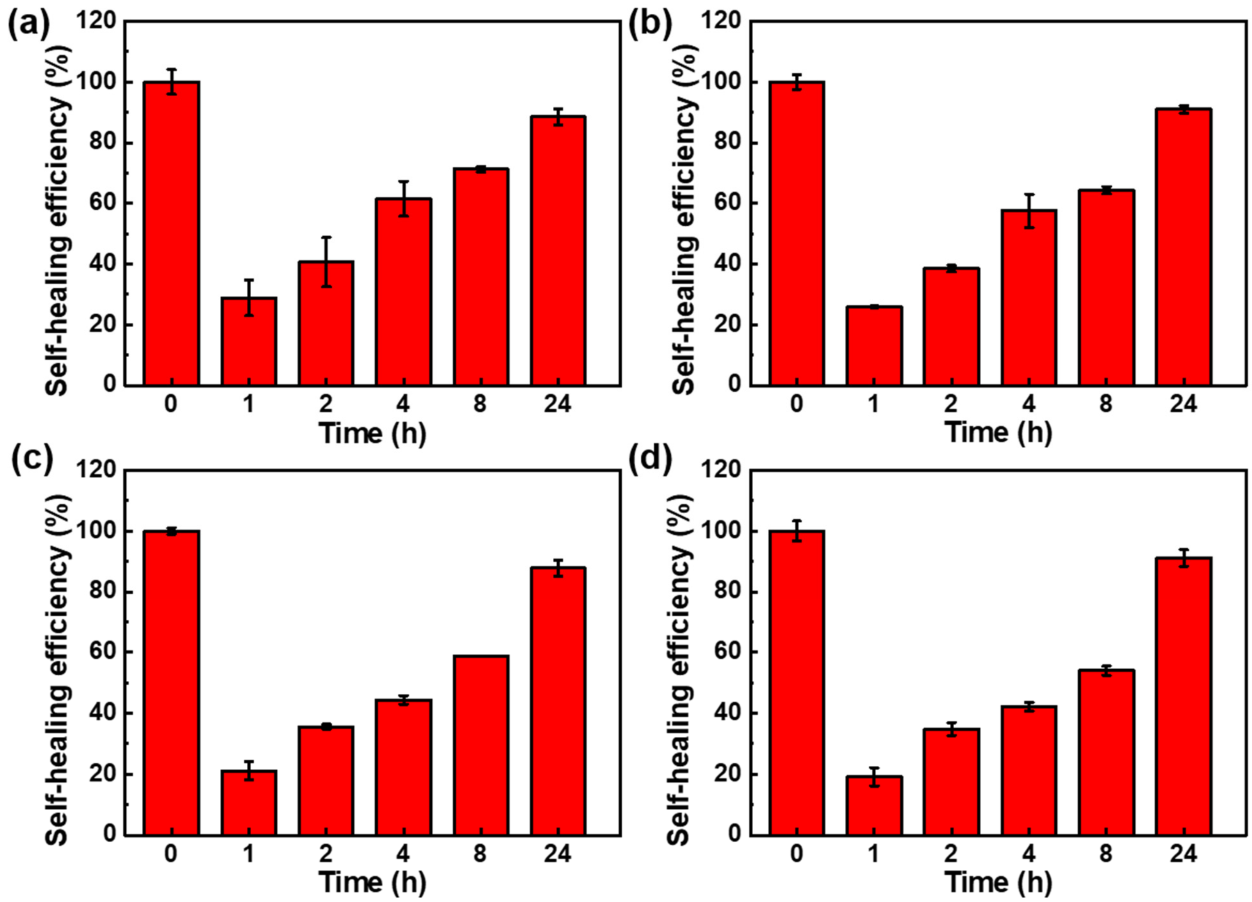

Figure S10. Self-healing efficiency of (a) PPG/ADA, (b) PPG/ADA-Sm^{3+}, (c) PPG/ADA-Eu^{3+}, and (d) PPG/ADA-Tb^{3+} hydrogels.

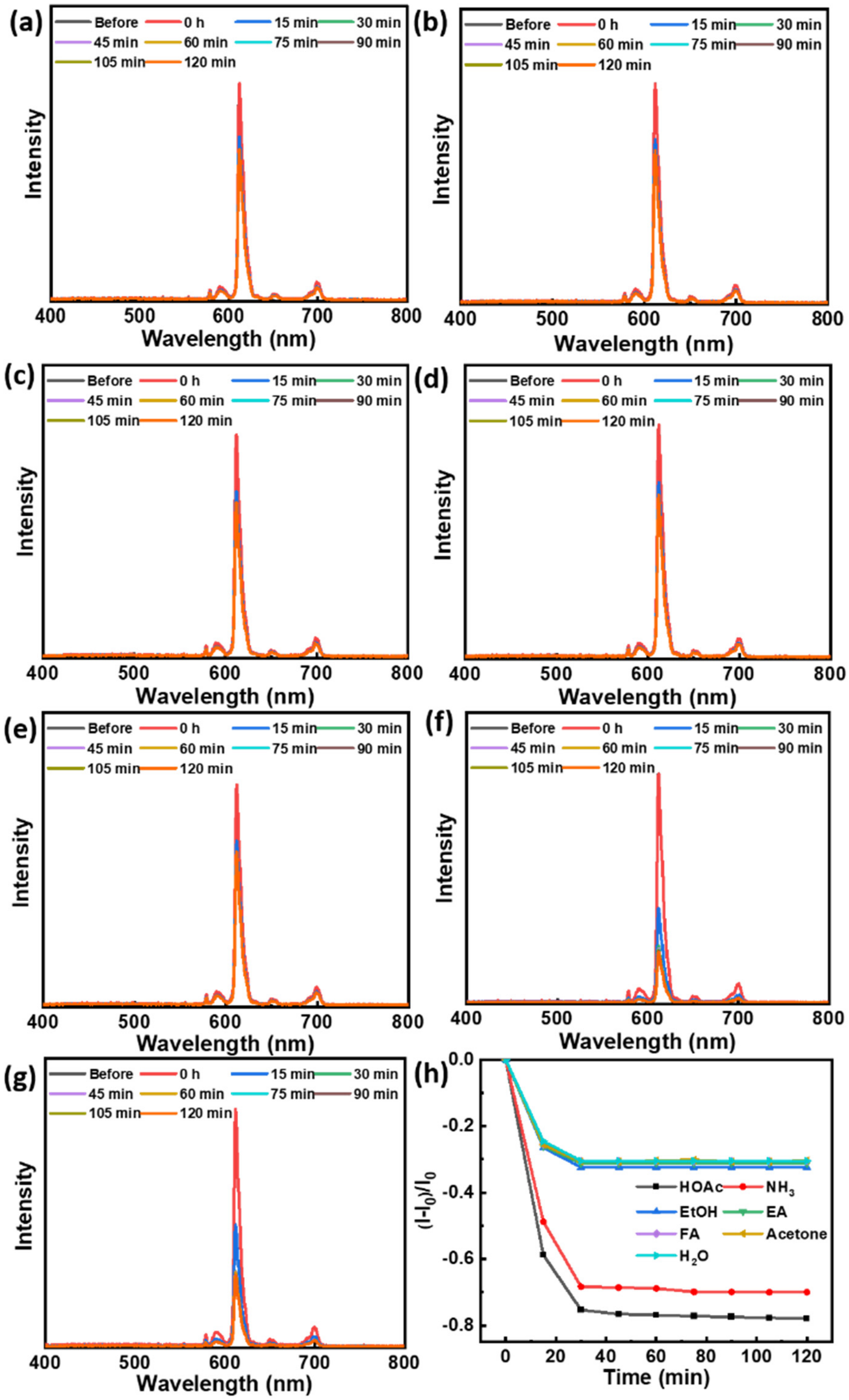

Figure S11. Luminescence spectra of PPG/ADA-Eu^{3+} lyophilized hydrogels exposed to various VOCs at different time points: (a) water, (b) acetone, (c) EA, (d) EtOH, (e) FA, (f) HOAc, and (g) NH_3. (h) Luminescence intensity changes ($I-I_0/I_0$) of PPG/ADA-Eu^{3+} lyophilized hydrogel exposed to various VOCs at different time points.

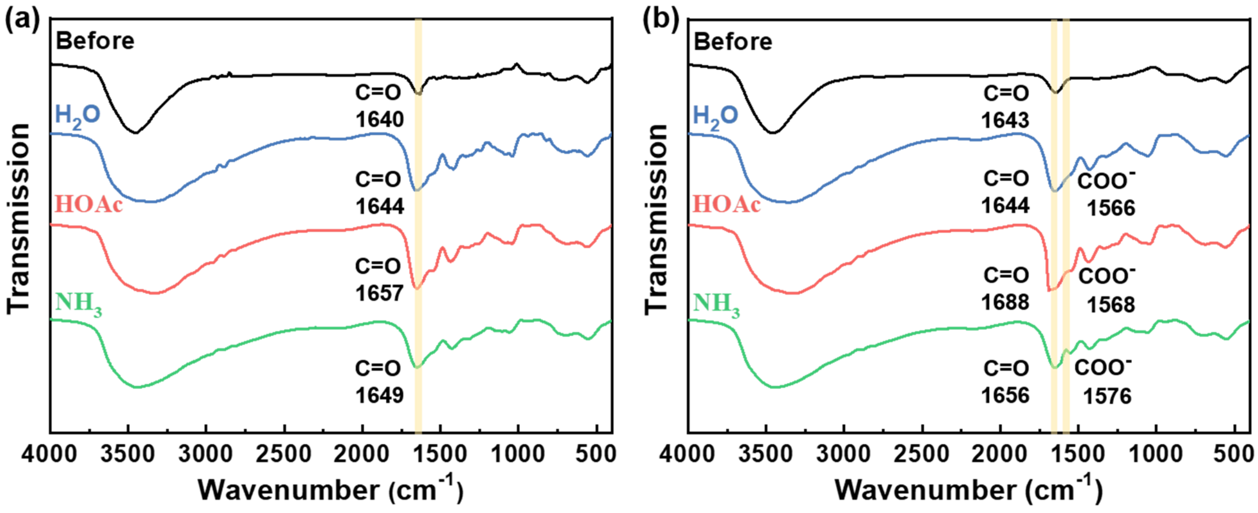

Figure S12. FTIR spectra of (a) PPG/ADA-Sm^{3+} and (b) PPG/ADA-Tb^{3+} lyophilized hydrogels before and after exposure to H_2O, HOAc, and NH_3 vapors for 2 hrs.

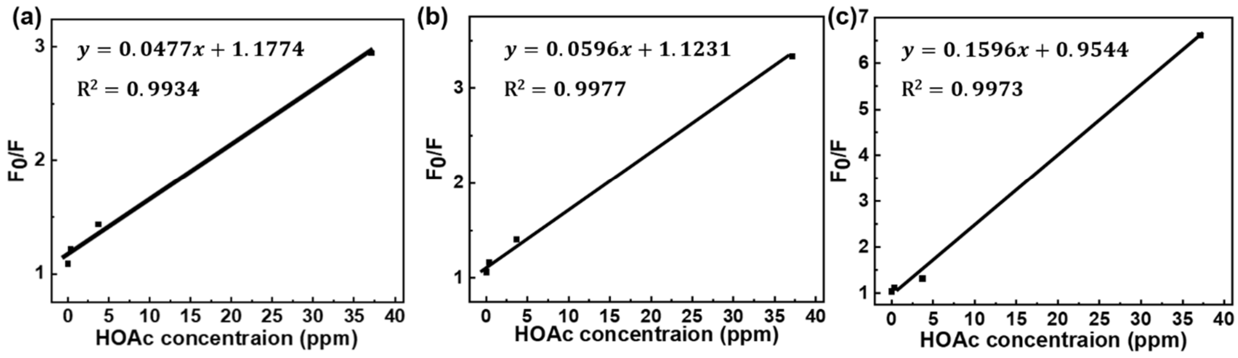

Figure S13. Fluorescence changes of (a) PPG/ADA-Sm^{3+}, (b) PPG/ADA-Eu^{3+}, and (c) PPG/ADA-Tb^{3+} lyophilized hydrogels exposed to HOAc vapor with varying concentrations. The concentrations of HOAc in the gas state were 37.15450, 3.72147, 0.37221, and 0.03722 ppm for the HOAc solutions with 0.1, 0.01, 0.001, and 0.0001 M, respectively, calculated based on the ideal gas law.

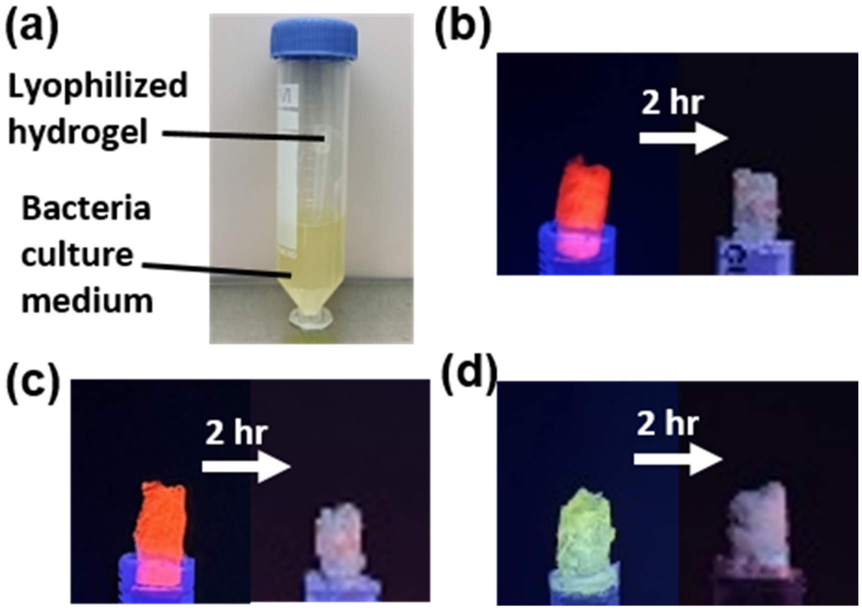

Figure S14. (a) Image of the setup for bacteria sensing. Luminescence quenching after sensing *S. aureus*: (b) PPG/ADA-Sm^{3+}, (c) PPG/ADA-Eu^{3+}, and (d) PPG/ADA-Tb^{3+} lyophilized hydrogels.

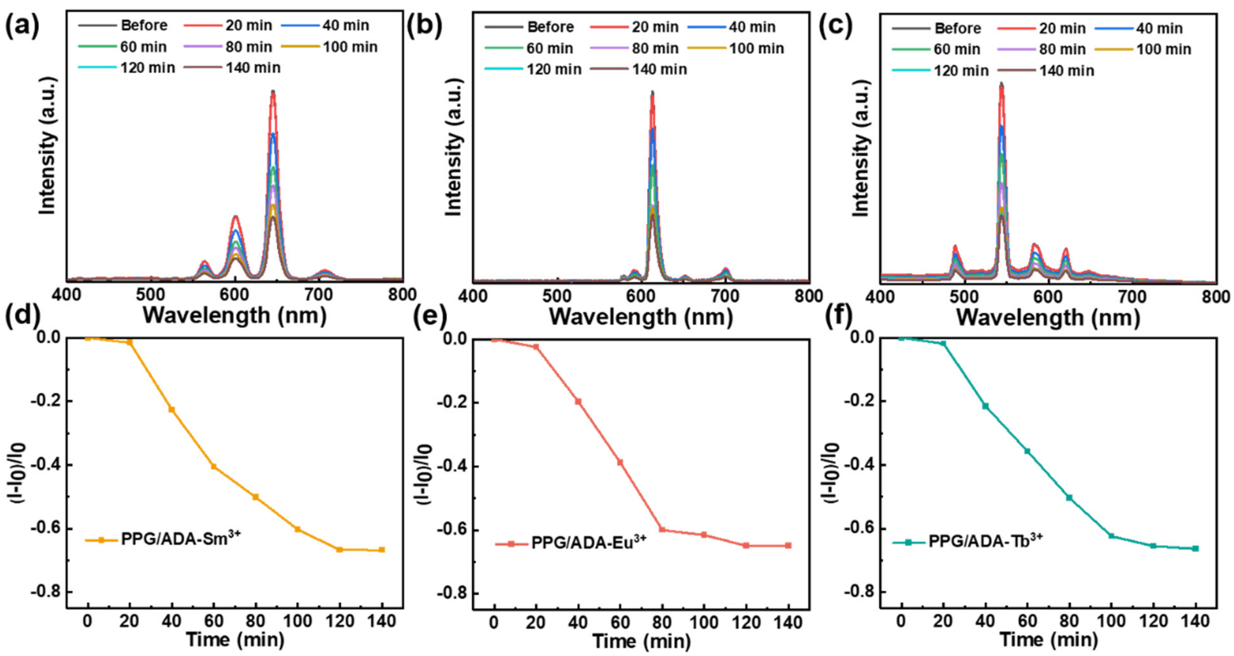

Figure S15. Luminescence spectra of PPG/ADA-Ln^{3+} lyophilized hydrogels exposed to *S.aureus* at different time points: (a) PPG/ADA-Sm^{3+}, (b) PPG/ADA-Eu^{3+}, and (c) PPG/ADA-Tb^{3+} lyophilized hydrogels. Luminescence intensity changes ($I-I_0/I_0$) of PPG/ADA-Ln^{3+} lyophilized hydrogels exposed to *S.aureus*: (d) PPG/ADA-Sm^{3+}, (e) PPG/ADA-Eu^{3+}, and (f) PPG/ADA-Tb^{3+} lyophilized hydrogels.

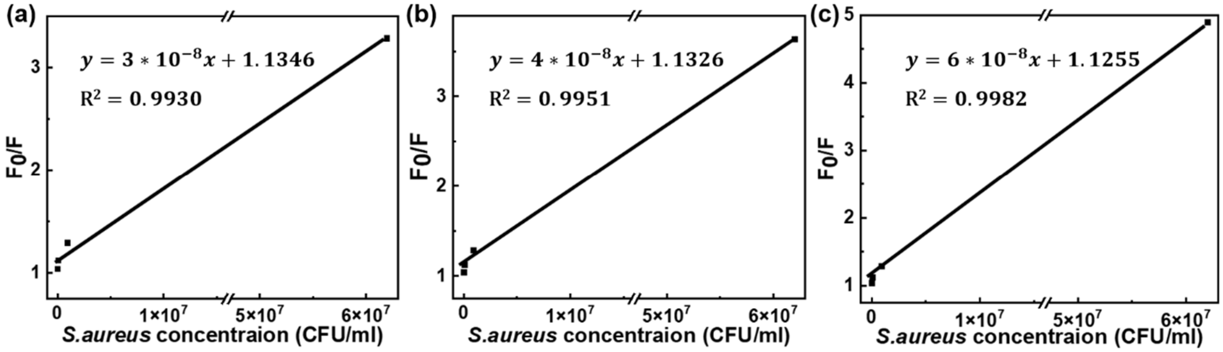

Figure S16. Fluorescence changes of (a) PPG/ADA-Sm^{3+}, (b) PPG/ADA-Eu^{3+}, and (c) PPG/ADA-Tb^{3+} lyophilized hydrogels exposed to *S. aureus* with varying concentrations after 2 hrs.

3. Table S1-S3

Table S1. Integration of the Ln-O peak in the XPS spectra of lyophilized hydrogels.

	**PPG/ADA-Sm^{3+}**	**PPG/ADA-Eu^{3+}**	**PPG/ADA-Tb^{3+}**
Position (eV)	532	531.8	532.0
Area	74800	129800	174900

Table S2. The pore size and porosity of lyophilized hydrogels were analyzed by micro-CT.

	**PPG/ADA**	**PPG/ADA-Sm^{3+}**	**PPG/ADA-Eu^{3+}**	**PPG/ADA-Tb^{3+}**
Average pore size (μm)	184.88	168.12	159.62	147.81
Closed porosity (%)	0.13	0.22	0.26	0.28
Open porosity (%)	88.49	81.84	82.61	89.65
Total porosity (%)	88.53	81.85	82.71	89.76

Table S3. Rheological analyses of hydrogels.

	**PPG/ADA**	**PPG/ADA-Sm^{3+}**	**PPG/ADA-Eu^{3+}**	**PPG/ADA-Tb^{3+}**
Storage modulus (kPa)	0.41 ± 0.01	7.94 ± 0.25	18.38 ± 0.30	29.14 ± 0.64
Loss modulus (kPa)	0.01 ± 0.05	1.62 ± 0.04	2.57 ± 0.06	5.43 ± 0.14
Crosslinking density (mole/m^3)	0.17 ± 0.01	3.37 ± 0 .10	8.06 ± 0.16	13.25 ± 0.22

4. References

(1) Shen, K.-H.; Chiu, T.-H.; Teng, K.-C.; Yu, J.; Yeh, Y.-C. Fabrication of triple-crosslinked gelatin/alginate hydrogels for controlled release applications. *Int. J. Biol. Macromol.* **2023**, *250*, 126133.

(2) Lou, C.; Yang, C.; Zheng, W.; Liu, X.; Zhang, J. Atomic layer deposition of ZnO on SnO_2 nanospheres for enhanced formaldehyde detection. *Sensors Actuators B: Chem.* **2021**, *329*, 129218.

(3) Xie, S.; Dai, T.; Li, F.; Liu, F.; Xu, Q.; Zhu, A.; Zhang, X. Shape memory luminescent cellulose/chitosan hydrogel for high sensitive detection of formaldehyde. *Int. J. Biol. Macromol.* **2023**, *233*, 123570.
